# Internal–External Heterogeneity in Downed Logs Across Decay Stages: Divergent Wood Chemistry and Contrasting Bacterial and Fungal Responses

**DOI:** 10.3390/microorganisms14091885

**Published:** 2026-08-25

**Authors:** Zhihao Chen, Yiqiong Zhang, Zhouchang Zhang, Tianjiao Song, Yufei Ji, Xinrui Li, Liangxuan Xu, Hua Yi, Yao Wang, Yanbing Lin

**Affiliations:** College of Life Sciences, Northwest A&F University, Yangling 712100, China; chenzhihao@nwafu.edu.cn (Z.C.); zhangyq0099@nwafu.edu.cn (Y.Z.); zhouchangzhang@nwafu.edu.cn (Z.Z.); 2023060644@nwafu.edu.cn (T.S.); jiyufei@nwafu.edu.cn (Y.J.); xinrui-li@nwafu.edu.cn (X.L.); xlx1111@nwafu.edu.cn (L.X.); yihua0406@nwafu.edu.cn (H.Y.); wangyao@nwsuaf.edu.cn (Y.W.)

**Keywords:** downed logs, microbial assembly, spatial heterogeneity, decay stages, bacterial and fungal communities, wood chemistry, cross-kingdom networks

## Abstract

Forests store carbon in downed logs, whose decomposition is driven by bacterial and fungal communities. Yet most studies treat individual logs as homogeneous units, obscuring internal–external gradients in wood chemistry as fine-scale habitat filters. To address this gap, we combined ALDEx2, SpiecEasi networks, Mantel tests and PLS-SEM on 24 samples from *Larix principis-rupprechtii* logs spanning three decay stages (I, III, and V) and two radial positions. Chemical heterogeneity between positions peaked at Stage III (5.9-fold difference in total nitrogen), with severe internal nitrogen and phosphorus depletion. Bacterial alpha diversity converged between positions by Stage V, whereas fungal communities maintained persistent divergence, and differentially abundant taxa showed stage- and position-associated enrichment patterns. Network topology did not differ detectably between internal and external wood. Path modelling showed contrasting associations: fungal composition tracked primarily the decay-stage axis, whereas bacterial composition was associated more weakly and mainly through an indirect nutrient channel. These results provide a fine-scale, descriptive account of divergent bacterial and fungal responses to within-log heterogeneity during downed-log decomposition in a temperate coniferous forest.

## 1. Introduction

Forests store more than 300 Gt of carbon globally, primarily as wood [1]. Downed logs are a major structural and functional component of forest ecosystems. They provide a long-lasting substrate that fuels microbial decomposition and drives nutrient return to the soil [2,3]. Bacteria and fungi are the principal biological engines of this process. Their community assembly and functional succession directly regulate the rate and pathway of carbon release from decaying wood [4,5]. Therefore, understanding the microbial ecology of downed log decomposition is essential for predicting forest carbon dynamics. In the temperate coniferous forests of the Qinling Mountains, downed logs of *Larix principis-rupprechtii* constitute a major carbon stock and have served as a model substrate for regional woody debris decomposition studies [6,7].

Traditionally, downed log decomposition has been viewed as a fungus-dominated process. Successional patterns from Ascomycota in early decay to Basidiomycota in intermediate and late stages are well documented [8,9,10]. However, recent studies show that bacteria are not merely passive early colonizers. They persist throughout decay and contribute directly to wood degradation through carbohydrate-active enzymes, nitrogen fixation, and organic matter mineralization [4,11,12]. Bacteria and fungi develop complex relationships spanning synergy, competition, and neutral co-existence [13,14]. These interactions jointly regulate decay rates and community functions [14,15,16]. Moreover, distinct fungal decay mechanisms create divergent chemical signatures that exert strong selection effects on associated bacterial communities [17,18]. Yet whether this filtering operates uniformly across the internal–external gradient remains untested.

Despite these advances, most studies still treat individual downed logs as homogeneous units. They rely on composite samples pooled from multiple drillings or saw cuts [5,19]. Yet downed logs are not uniform substrates. External wood near the bark surface and internal wood near the heartwood differ fundamentally in moisture penetration, oxygen diffusion, nutrient leaching, and the relative proportions of lignin and cellulose [8,14]. These inherent gradients generate contrasting microenvironments that could act as powerful habitat filters on microbial colonization [20]. Recent work on trees has shown that heartwood and sapwood harbor distinctly partitioned microbial communities. Heartwood represents a unique niche enriched in specialized bacteria and archaea [21]. In deadwood, fine-scale sampling reveals that bacterial communities show strong spatial heterogeneity within a single log, whereas fungal communities are more evenly distributed [19]. Whether similar internal–external partitioning exists across different decay stages, and how within-log microenvironmental heterogeneity relates to microbial community assembly remains poorly understood. This gap limits our ability to determine whether microorganisms respond to local chemical gradients within a log or merely to the coarse proxy of decay stage. Beyond documenting such spatial partitioning, a deeper question is whether bacterial and fungal responses to these gradients reflect shared environmental sensitivity, direct or indirect cross-kingdom interactions, or a combination of both [22].

Current literature often portrays bacterial priming and fungal succession as a tightly coupled metabolic relay [5,17,22]. While co-occurrence patterns have been widely used to infer such cross-kingdom interactions [23], significant spatial associations may simply reflect shared environmental sensitivity rather than true ecological interaction [24]. At the level of hypothesised functional groups, it remains unclear whether bacterial polysaccharide depolymerizers and fungal lignocellulose degraders establish direct metabolic partnerships, or whether they respond independently to the same chemical template shaped by wood decay. Both scenarios are ecologically plausible: fungal hyphae extend throughout the deadwood and can transport nutrients across the substrate [19]; distinct fungal decay mechanisms create different chemical signatures that select for different bacterial taxa [17]; and historical contingency and priority effects can loosen any tight metabolic coupling [25]. Critically, observational association data alone cannot distinguish shared environmental responses from direct or indirect cross-kingdom interactions [24]. Delineating where these possibilities lie is therefore essential for understanding the microbial drivers of downed log decomposition.

To bridge these gaps, we adopted a multi-scale analytical framework combining scale-aware differential abundance profiling, cross-kingdom association network inference, distance-based community–environment analysis, and path modelling (Section 2.6). The most responsive taxa were organised into enrichment groups defined solely by their enrichment patterns.

Against this background, the present study was designed to test internal–external gradients within downed logs. The main objectives were: (1) to characterise internal–external heterogeneity in wood chemistry and microbial communities across decay stages; (2) to compare how bacterial and fungal communities respond to these within-log gradients; and (3) to examine whether decay stage and position are associated with bacterial and fungal community composition through shared chemical pathways.

## 2. Materials and Methods

### 2.1. Study Area

This study was conducted at the Huoditang Experimental Forest Farm of Northwest A&F University. The site is located on the southern slope of the central Qinling Mountains in Ningshan County, Shaanxi Province, China (33°18′–33°28′ N, 108°21′–108°39′ E). The forest covers 2037 hm^2^ with elevations ranging from 800 to 2500 m. Slopes typically vary between 20° and 50°.

The region has a warm temperate climate. Mean annual temperature ranges from 8 to 10 °C. Annual precipitation varies between 900 and 1200 mm, while annual evaporation ranges from 800 to 950 mm. The frost-free period lasts approximately 170 days. The soils in the study area are classified as mountain brown soils, typical of the Qinling Mountains. The vegetation at the Huoditang Experimental Forest Farm is dominated by mixed temperate coniferous and broadleaf forests, among which *Larix principis-rupprechtii* is a primary constituent and the focal tree species of regional forest ecological research [6,7].

### 2.2. Log Sampling and Experimental Design

Field sampling was carried out in July 2025. We first conducted a preliminary survey to identify the most representative downed logs. Logs with a diameter at breast height (DBH) of 25 ± 5 cm constituted the majority of fallen wood in the forest stands. Therefore, we selected logs within this size class that were in contact with the ground and had similar site conditions as our research subjects.

We divided the log decomposition process into three stages (I, III, and V) [5] based on the structural characteristics of internal and external wood tissues [26]. Stage I represents the initial phase with minimal decomposition. Stage V represents the final phase with advanced decomposition. For each decay stage, we randomly selected four replicate logs. Within this five-class system [5,26], we sampled classes I, III, and V to represent the initial, intermediate, and final phases of decomposition, thereby spanning the full decay continuum with the maximal structural contrast between alternate classes. Importantly, Qi et al. (2023) suggested that simplifying the five decay stages into three representative stages may be a more suitable strategy for downed log microbial research, as microbial community structure and function are highly analogous between early (I) and late decay stages (IV, V), while the intermediate stages present distinct microbial characteristics [5]. The intermediate class III was considered indispensable because microbial diversity and community divergence at this site were previously found to be greatest at intermediate decay.

From each log, we collected wood samples from two distinct spatial positions. The external position was located 0–2 cm beneath the bark surface. The internal position was located 8–10 cm deep from the surface, toward the log center. This design captured the contrasting microenvironments between the outer and inner wood tissues. The sampling strategy yielded a total of 24 samples (3 decay stages × 2 spatial positions × 4 replicates).

We used different sampling methods depending on the decay stage. For logs in Stage I with intact wood structure, we extracted wood cores using a cordless drill equipped with a wood auger. To prevent cross-contamination between samples, we cleaned the drill bit sequentially with sterile distilled water and 70% ethanol prior to sampling each new log. For heavily decomposed logs in Stages III and V, where the wood structure was too soft for drilling, external and internal materials were collected from the corresponding depths directly into sterilized aluminum boxes.

The choice of sampling tool was dictated by the physical integrity of the wood at each decay stage, following the established downed-log protocol used at this site [5]: intact (Stage I) logs could be cored, whereas softened (Stage III and V) logs could not maintain core structure and were sampled directly. Within each decay stage, the paired internal and external samples of every log were collected with the same tool.

All collected samples were immediately placed in sterile sampling bags. They were transported to the laboratory on ice within 4 h of collection. Upon arrival, samples were stored at −80 °C until further processing. Before DNA extraction and chemical analysis, we ground the samples in a grinder with liquid nitrogen at a frequency of 30 Hz for 2 min. The resulting wood powder was divided into two portions. One portion was used for total DNA extraction for molecular analysis. The other portion was used for determining the physicochemical properties of the downed logs.

### 2.3. Chemical Analysis

The contents of cellulose, hemicellulose, and lignin were quantified using the Van Soest fiber analysis with sequential detergent digestion [27]. Neutral detergent fiber (NDF), acid detergent fiber (ADF), and acid detergent lignin (ADL) were determined from approximately 1.0 g of dried sample. Hemicellulose content was calculated as NDF minus ADF, cellulose content as ADF minus ADL, and lignin content as ADL minus acid-insoluble ash.

For total nitrogen (TN), phosphorus (TP), and potassium (TK) analyses, approximately 0.1 g of ground wood sample was digested using the sulfuric acid-hydrogen peroxide (H_2_SO_4_-H_2_O_2_) method [28]. TN was subsequently determined by the indophenol blue colorimetric method at 625 nm [29]. TP was measured by the molybdenum-antimony-ascorbic acid colorimetric method at 880 nm [30], and TK was analyzed by flame photometry [31]. All chemical analyses were performed in triplicate, with results expressed on a dry weight basis.

### 2.4. DNA Extraction and Amplicon Sequencing

Total genomic DNA was extracted from 25 mg of homogenized sawdust using the DNeasy PowerSoil Pro Kit (Qiagen, Hilden, Germany) according to the manufacturer’s protocol. For bacterial community analysis, the V5–V7 hypervariable regions of the 16S rRNA gene were amplified using the forward primer 799F AACMGGATTAGATACCCKG and the reverse primer 1193R ACGTCATCCCCACCTTCC. For fungal community analysis, the ITS1 region was amplified using the forward primer ITS1F CTTGGTCATTTAGAGGAAGTAA and the reverse primer ITS2 GCTGCGTTCTTCATCGATGC. PCR reactions were performed using Phusion High-Fidelity PCR Master Mix (New England Biolabs, Ipswich, MA, USA) with the following cycling conditions: initial denaturation at 98 °C for 1 min; followed by 30 cycles of 98 °C for 10 s, 50 °C for 30 s, and 72 °C for 30 s; and a final extension at 72 °C for 5 min. The PCR products were purified using magnetic beads, quantified with a Qubit fluorometer (Invitrogen, Carlsbad, CA, USA), and pooled in equimolar concentrations. Sequencing libraries were prepared with index adapters and sequenced on the NovaSeq 6000 platform (Illumina, San Diego, CA, USA) to generate 250 bp paired-end reads.

### 2.5. Sequence Data Processing

Raw paired-end reads were processed using QIIME 2 (version 2024.2) for demultiplexing, quality filtering, and generation of amplicon sequence variants (ASVs) via the DADA2 plugin [32]. Taxonomic classification was performed using pre-trained classifiers against the SILVA 138 SSURef NR99 database [33] and UNITE 9.0 database [34]. Mitochondrial, chloroplast, and non-fungal eukaryotic sequences were removed using custom filtering scripts. In total, 20 chloroplast/mitochondrial ASVs (752 reads) were removed from the 16S dataset; no non-fungal sequences were detected in the ITS dataset. The raw amplicon sequencing reads generated in this study have been deposited in the NCBI Sequence Read Archive under BioProject accession number PRJNA1499194; per-sample BioSample and SRA run accessions are listed in Table S12.

### 2.6. Bioinformatics and Statistical Analyses

To standardize sequencing depth, ASV tables were rarefied in R to the minimum library size (30,120 reads per sample for bacteria; 54,900 for fungi; random seed 42); unrarefied count tables were retained for differential abundance analysis (see below). Alpha diversity (Chao1 richness, observed ASVs, Shannon index with natural logarithm, and Pielou’s evenness) and beta diversity were calculated using the phyloseq (version 1.54.2) [35] and vegan (version 2.7-3) [36,37] packages in R (version 4.5.3). Wood physicochemical properties and alpha diversity indices were analysed with linear mixed models, value ~ Stage × Position + (1 | Log_ID), fitted by REML with Type III tests (Satterthwaite denominator df) and Benjamini–Hochberg adjustment; planned Internal-vs-External contrasts within each stage were obtained from estimated marginal means with Holm adjustment. For beta diversity, normalized weighted UniFrac distances (Lozupone normalization) were used for bacteria, whereas Bray–Curtis distances were used for fungi, because the ITS1 region does not support reliable phylogenetic tree inference. Community differences were assessed by PERMANOVA (999 permutations) under permutation schemes respecting the paired design: Stage was tested by permuting intact logs among decay stages, and Position and the Stage × Position interaction by swapping positions within logs. Main effects were taken from the additive model and the interaction from the full model; pairwise comparisons used exact enumeration of all permissible permutations. Homogeneity of multivariate dispersions was checked with betadisper.

Differential abundance analysis was performed on unrarefied, genus-level aggregated counts using ALDEx2 (version 1.42.0) with the Scale Model (γ = 0.5) and 1000 Monte Carlo samples [38], retaining genera with relative abundance >5 × 10^−4^ in at least four samples. Effect sizes (standardized median difference) were calculated for 18 pairwise comparisons (spatial: Internal vs. External at decay stages I, III and V, using Wilcoxon tests paired by log identity; temporal: Stage I vs. III, I vs. V, and III vs. V within each position, using Welch’s *t*-test), with FDR correction (we.eBH < 0.05; we.eBH < 0.10 reported as a trend for bacteria).

Cross-kingdom co-occurrence networks were inferred at the genus level from the same unrarefied count tables used for differential abundance analysis. Bacterial (237 genera) and fungal (128 genera) counts were merged into a single cross-kingdom matrix (365 genera). Three sample sets were analysed: all samples (*n* = 24), internal wood (*n* = 12), and external wood (*n* = 12). Within each set, genera were retained when detected in at least 25% of samples, yielding 355, 317, and 356 genera, respectively. To make the two positional networks directly comparable, the Internal and External networks were constructed as a pair restricted to the 308 genera passing the prevalence threshold in both sets, with samples ordered so that the internal and external samples of the same log occupied the same row position. Networks were inferred with SpiecEasi (version 1.1.2; method = ‘mb’, i.e., neighbourhood selection) [39] through the NetCoMi workflow (version 1.2.0) [40], using StARS model selection (lambda.min.ratio = 0.1, nlambda = 30, subsample.ratio = 0.8, rep.num = 20). No additional zero replacement or normalisation was applied before inference. Topological properties were computed with netAnalyze; nodes were classified as module hubs (Zi > 2.5), connectors (Pi > 0.62), or network hubs (both criteria) following Olesen et al. [41]. The two positional networks were compared with netCompare using 500 permutations that exchange the paired internal and external samples within each log, across ten global and comparative measures with Benjamini–Hochberg adjustment. StARS stability values were ≤0.05 for all three networks (Table S9). Node and edge tables were exported to Gephi 0.10.1 for visualisation [42].

Differentially abundant genera were organized into enrichment groups separately for each kingdom. Genera flagged in the internal-wood temporal comparisons (Stage I vs. III, I vs. V, and III vs. V; we.eBH < 0.10 for bacteria and <0.05 for fungi) were assigned to bacterial (B-EG-I, B-EG-III, B-EG-V) or fungal (F-EG-I, F-EG-III, F-EG-V) enrichment groups according to the decay stage toward which they were enriched. Internal wood was used as the reference habitat for group definition, because it is the position where the within-log chemical divergence is expressed; a single position kept the bacterial and fungal groups defined under one identical rule. Genera enriched toward more than one stage received multiple memberships, and taxonomically unclassified genera were retained. Paired position comparisons and external-wood comparisons were not used for group assignment. Group memberships are reported in Table S8. Functional annotations from FUNGuild (fungi) [43] and FAPROTAX [44] together with the published literature (bacteria) were used only for post hoc ecological interpretation of the groups (Section 4.3), not for their definition.

Associations between microbial community structure and wood chemistry were evaluated with Mantel tests at two levels. As a selection-free baseline, we used the whole-community distance matrices from the beta-diversity analysis directly (normalized weighted UniFrac for bacteria; Bray–Curtis for fungi). For each enrichment group, we computed a group-level distance matrix as Bray–Curtis dissimilarity on the relative abundances of its member genera (member reads divided by total library size). We computed chemical distances as Euclidean distances on z-score-standardized values of each of the eight properties separately. Mantel statistics were computed as Spearman’s rank correlations between distance matrices. Because group membership was defined by decay-stage enrichment, we assessed significance under three permutation schemes that respect the paired design: (i) permuting intact logs within decay stages, enumerating all ${\left(4!\right)}^{3}$ − 1 = 13,823 permissible permutations (primary inference, separating associations beyond the decay-stage axis on which group membership was defined); (ii) swapping the internal and external samples within each log, enumerating all $2^{12}$ − 1 = 4095 swaps; and (iii) freely permuting intact logs (9999 random permutations, seed 42) as a nominal reference. We computed one-sided *p*-values and applied Benjamini–Hochberg adjustment to the primary within-stage *p*-values within each analysis section [45].

Partial least squares path modelling (PLS-SEM) was used to evaluate direct and indirect pathways linking decay stage, spatial position, wood chemistry, and microbial community composition, using the plspm package (version 0.6.0) [46,47]. Two structurally identical models were specified, one per kingdom (M1, bacteria; M2, fungi). The decay stage was entered as two standardized dummy variables with Stage I as the reference, and position as a single dummy variable (Internal = 0, External = 1); all observed variables, including the dummies, were z-score standardized, and models were fitted with scaled = FALSE. Three wood-chemistry mediators were specified: carbon polymers (cellulose + hemicellulose), lignin (single indicator), and nutrient availability (TN + TP + TK). Structural carbon fractions and lignin were expressed as absolute contents on a fresh-wood basis (g/kg), computed from each fraction’s percentage of dry matter and the sample dry-matter fraction, to avoid closure artefacts among compositional percentages; TN, TP, and TK entered as measured absolute contents. Dry matter and ash were not entered as mediators, because dry matter served as the conversion base and ash represents incombustible mineral residue. The outcome was the sample score on the first PCoA axis of community composition (normalized weighted UniFrac for bacteria; Bray–Curtis for fungi), with larger scores corresponding to more advanced decay [48]. Measurement quality was assessed by outer loadings (≥0.70), average variance extracted (>0.50), composite reliability, cross-loadings, and heterotrait–monotrait ratios (<0.85), and overall fit by the goodness-of-fit index (GoF) [49,50]. Because the internal and external samples of a log are not independent, inference relied on cluster bootstrap resampling of the 12 logs (1999 replicates; 95% percentile confidence intervals), with an explicit row-wise bootstrap (1000 replicates) as a comparison. Collinearity was quantified by variance inflation factors computed on latent variable scores, under a pre-registered rule (VIF > 5) to re-estimate a reduced model without the lignin mediator; both full and reduced estimates are reported. Sensitivity analyses comprised within-log difference models (external minus internal, *n* = 12) and models using the second PCoA axis as the outcome. All measurement, validity, collinearity, path, effect, and sensitivity statistics are reported in Table S11.

## 3. Results

### 3.1. Internal–External Divergence in Wood Chemistry and Microbial Communities Across Decay Stages

**Figure 1:**
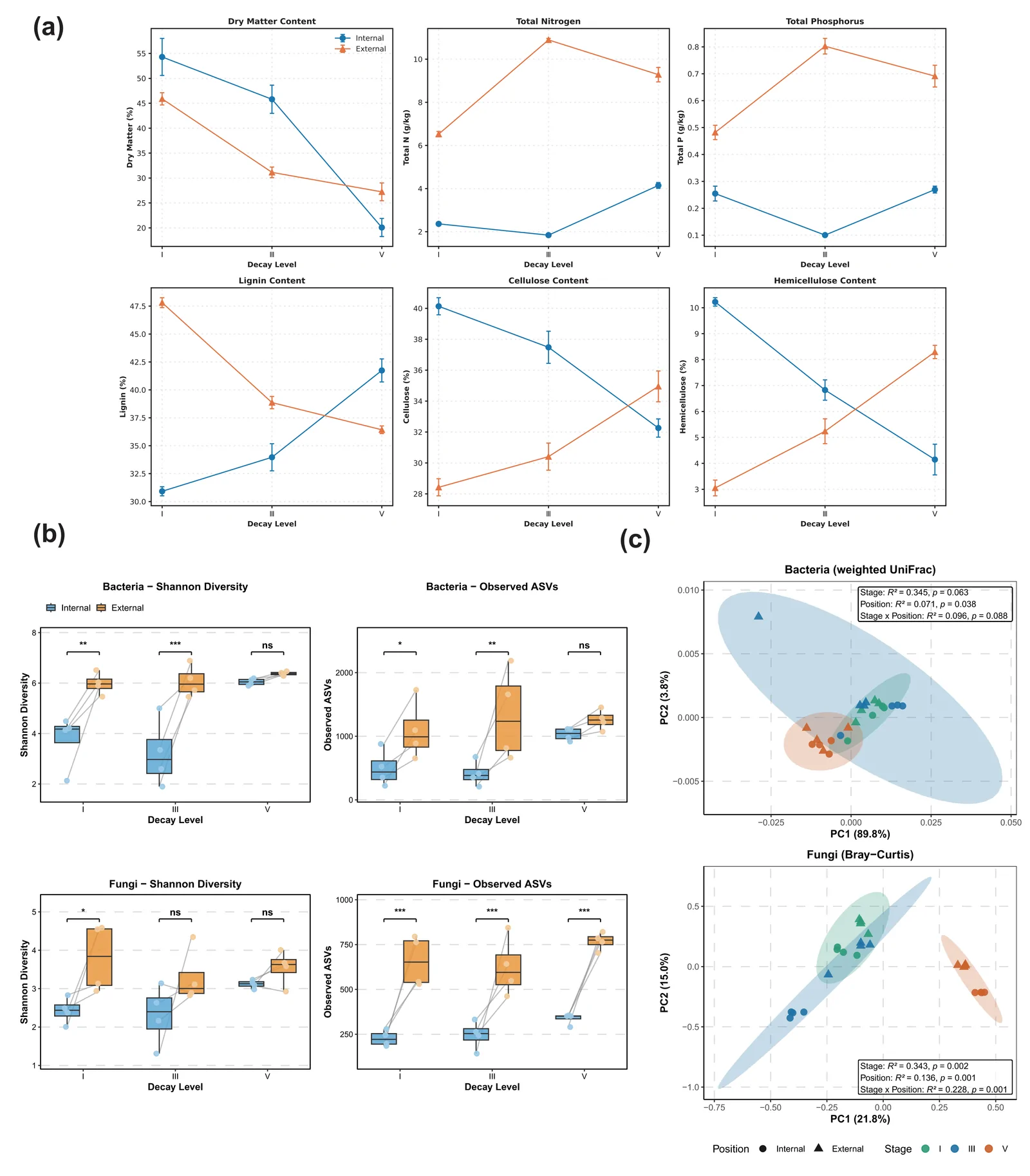
Wood chemistry, alpha diversity, and community structure across decay stages (I, III, V) and positions. (**a**) Wood dry matter, total nitrogen, total phosphorus, lignin, cellulose, and hemicellulose (mean ± SE, *n* = 4; Internal: blue; External: orange). Linear mixed models with log identity as a random intercept showed significant Stage × Position interactions for all eight measured properties after Benjamini–Hochberg adjustment ($p_{BH}$ < 0.01; Table S2). (**b**) Bacterial and fungal alpha diversity (Shannon index and observed ASVs). Boxplots show individual samples n=4; grey lines connect paired samples from the same log. Brackets indicate planned Internal-vs-External contrasts from the mixed models (Holm-adjusted): * *p* < 0.05, ** *p* < 0.01, *** *p* < 0.001, ns, not significant (Tables S3–S5). (**c**) PCoA based on normalized weighted UniFrac distances (bacteria) and Bray–Curtis distances (fungi). Points are colored by decay stage and shaped by position (circles: Internal; triangles: External); ellipses show 95% confidence ellipses by stage. Insets report PERMANOVA results under a paired permutation design (999 permutations; Table S6).

Downed log decomposition created divergent chemical microenvironments between internal and external wood (Figure 1a; Table S1). Linear mixed models with log identity as a random intercept confirmed significant Stage × Position interactions for all chemical properties after Benjamini–Hochberg adjustment ($p_{BH}$ < 0.01; Table S2) (model $R^{2}$ in Figure S1). Nutrient and mineral gradients between positions were largest at Stage III, whereas structural carbon fractions reversed their radial patterns across decay stages. Dry matter declined continuously throughout decay, and internal wood showed a steeper drop from Stage III onward as moisture progressively penetrated the log interior. Total nitrogen and total phosphorus peaked in external wood at Stage III (TN = 10.89 g/kg) but remained low in internal wood (TN = 1.84 g/kg), creating a 5.9-fold disparity. Lignin proportion increased internally (30.9% to 41.7%) but decreased externally (47.8% to 36.4%); cellulose and hemicellulose showed the opposite pattern.

Microbial alpha diversity responded asymmetrically to this chemical gradient (Figure 1b; Tables S3–S5). Bacterial Shannon diversity differed significantly by stage, position, and their interaction (Stage × Position $p_{BH}$ = 0.025; Table S5). External wood maintained higher bacterial diversity at Stages I and III, whereas the internal–external difference was no longer detectable at Stage V. In contrast, fungal observed ASVs remained 2.3–2.9-fold higher in external than internal wood at all three stages (Position $p_{BH}$ < 0.001), with no recovery in late decay.

Beta diversity revealed contrasting assembly patterns (Figure 1c; Table S6). Bacterial communities were structured by spatial position ($R^{2}$ = 0.071, p = 0.038), with a large but non-significant decay-stage effect ($R^{2}$ = 0.345, p = 0.063) and a non-significant Stage × Position interaction ($R^{2}\;$= 0.096, p = 0.088). Bacterial composition differed neither between Stages I and III (p = 0.914) nor between III and V overall (p = 0.143); the III–V divergence was detectable only within internal wood ($R^{2}$ = 0.710, *p* = 0.029). Fungal communities differed between all stage pairs (exact p = 0.029, $p_{BH}$ = 0.037) and showed a significant Stage × Position interaction ($R^{2}$ = 0.228, p = 0.001), with stronger compositional shifts in internal than external wood. The internal–external effect size increased monotonically from Stage I ($R^{2}$ = 0.403) to III (0.563) and V (0.812), although exact within-stage tests are limited by the number of logs (minimum achievable p = 0.0625). Fungal dispersion also differed among stages (betadisper p = 0.024), whereas bacterial dispersions were homogeneous.

Together, these results identify Stage III as the point of maximum nutrient and mineral heterogeneity within downed logs and show that bacterial and fungal communities followed contrasting spatial patterns. Bacterial internal–external differences were no longer detectable at Stage V, whereas fungi showed continuous turnover and increasingly pronounced spatial divergence.

### 3.2. Microbial Community Composition Across Decay Stages

Bacterial communities were dominated by the *Burkholderia-Caballeronia-Paraburkholderia* complex across all decay stages. This group showed strong spatial heterogeneity, particularly at Stage III, where it reached 56.0% in internal wood but remained at 14.1% in external wood. Other abundant bacterial genera included *Pseudomonas*, *Sphingomonas*, and unclassified Micropepsaceae, with *Pseudomonas* being more prevalent in internal wood at early stages (14.6% at Stage I) and declining thereafter.

Fungal communities displayed a clear phylum-level succession from Ascomycota-dominated early stages to Basidiomycota-dominated late stages. At Stage I, Ascomycota accounted for 77.2% (external) and 60.9% (internal) of the community, while Basidiomycota represented only 20.0% and 22.4%, respectively. This pattern reversed at Stage III, where Basidiomycota increased to 78.1% (internal) and 52.3% (external), and remained dominant through Stage V (68.1–73.0%). Detailed taxonomic composition at the bacterial genus and fungal phylum levels is provided in Figure S2.

These contrasting patterns suggest that bacteria and fungi respond differently to wood decay processes. Bacterial assembly showed stage-specific spatial divergence, particularly driven by the heterogeneous distribution of the *Burkholderia-Caballeronia-Paraburkholderia* complex. Fungal assembly exhibited consistent directional turnover, with Basidiomycota progressively replacing Ascomycota across all spatial positions.

### 3.3. Differentially Abundant Taxa Show Stage- and Position-Associated Enrichment Patterns

#### 3.3.1. Bacterial Enrichment Is Concentrated in Stage III Internal Wood

**Figure 2:**
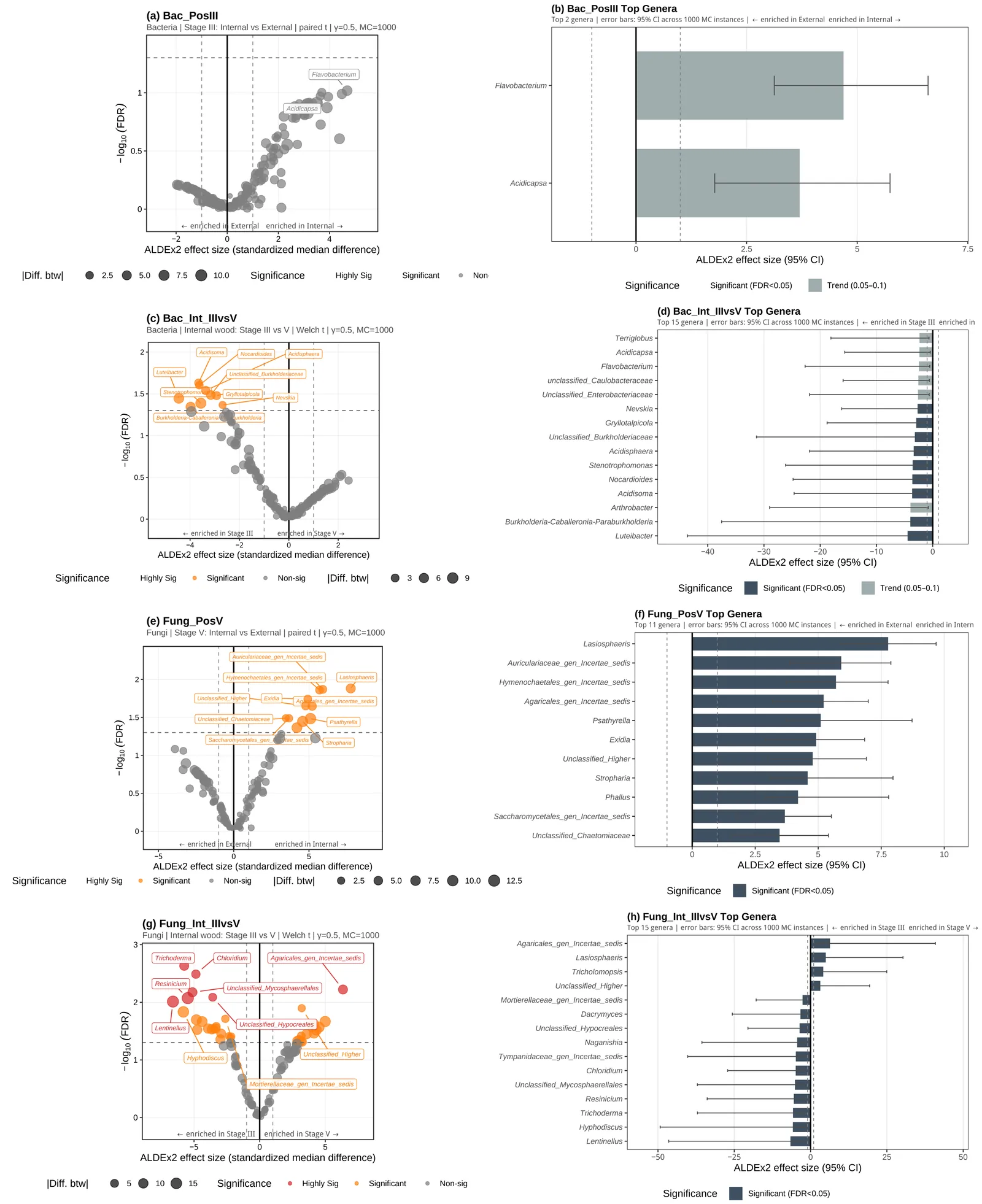
Differential abundance of bacterial and fungal genera (ALDEx2; Section 2.6). (**a**,**c**,**e**,**g**) Volcano plots of effect size versus −log10(FDR). (**b**,**d**,**f**,**h**) Effect sizes with 95% Monte Carlo intervals for top-ranked genera. In all panels, arrows indicate enrichment direction; dashed lines mark the significance threshold (FDR = 0.05) in volcano plots and effect sizes of −1 and 1 (between-group difference equal to within-group variation) in volcano, effect, and MA plots. (**a**,**b**) Internal vs. External bacteria at Stage III; (**c**,**d**) Stage III vs. V bacteria within internal wood; (**e**,**f**) Internal vs. External fungi at Stage V; (**g**,**h**) Stage III vs. V fungi within internal wood. All 18 pairwise comparisons are summarised in Table S7; MA plots for the four featured comparisons are provided in Figure S3 and volcano plots for twelve further comparisons in Figure S4.

Bacterial differential abundance was concentrated in internal wood and pointed away from late decay. Stage III internal wood was enriched in the *Burkholderia-Caballeronia-Paraburkholderia* complex (56.0%), *Gryllotalpicola*, and *Luteibacter* (Figure 2c,d; Table S7), whereas *Pseudomonas* was enriched in Stage I internal wood. *Acidisoma* was likewise enriched in Stage III internal wood. Notably, no bacterial genus was enriched toward Stage V in any comparison. No bacterial genus differed significantly between internal and external wood at any decay stage (I, III, or V) after paired testing (Figure 2a,b; Table S7), and no significant genus was detected in any external-wood comparison; bacterial differentiation was thus confined to internal wood.

#### 3.3.2. Fungal Enrichment Spans All Decay Stages and Is Directed Toward Internal Wood

Fungal communities exhibited clear stage-associated shifts (Figure 2e–h; Table S7). *Botryobasidium* was enriched in early internal decay. Stage III internal wood was characterized by the enrichment of *Resinicium* and *Lentinellus* (effect sizes −5.48 and −6.61 in the III-vs-V comparison, respectively), together with *Trichoderma* and *Mycena*. Stage V internal wood was enriched in *Lasiosphaeris*, *Stropharia*, and *Phallus*; these three genera were also significantly enriched in internal relative to external wood in the paired comparison at this stage (effects +7.78, +4.58, and +4.20, respectively; Figure 2e,f). *Dacryopinax* and *Tricholomopsis* were likewise enriched toward Stage V.

#### 3.3.3. Enrichment Group Assignment

To organize the differentially abundant genera for downstream co-occurrence network analyses and for the group-level characterisation reported in the Supplementary Materials, we assigned them to enrichment groups based solely on their enrichment patterns in the internal-wood temporal comparisons (Section 2.6). Each kingdom yielded three groups, corresponding to enrichment toward decay Stage I, III, or V (Table S8). The bacterial groups comprised 9 (B-EG-I; e.g., *Pseudomonas*, *Sphingomonas*, *Microbacterium*), 21 (B-EG-III; e.g., the *Burkholderia-Caballeronia-Paraburkholderia* complex, *Luteibacter*, *Gryllotalpicola*, *Acidisoma*, *Nocardioides*), and 0 genera (B-EG-V): no bacterial genus was enriched toward Stage V. The fungal groups comprised 15 (F-EG-I; e.g., *Botryobasidium*, *Armillaria*, *Scytalidium*, *Penicillium*), 21 (F-EG-III; e.g., *Resinicium*, *Lentinellus*, *Mycena*, *Chloridium*, *Exophiala*), and 15 genera (F-EG-V; e.g., *Lasiosphaeris*, *Stropharia*, *Phallus*, *Dacryopinax*, *Tricholomopsis*, *Podospora*). Ten genera whose enrichment patterns spanned two stages were assigned to both corresponding groups (e.g., *Trichoderma* to F-EG-I and F-EG-III). Genera whose differential abundance was restricted to external wood or to position contrasts alone were not assigned to any group. The external-wood temporal comparisons flagged additional fungal genera, which remain fully reported in Table S7; the groups were kept within internal wood so that they characterise stage-associated enrichment in the habitat where the within-log filter operates. In the paired position comparisons, all significantly position-associated fungal genera were enriched in internal wood (4 genera at Stage I and 11 genera each at Stages III and V), whereas no bacterial genus showed significant position-associated enrichment. These groups are empirical, enrichment-based groupings; their ecological interpretation is presented in Section 4.3.

### 3.4. Cross-Kingdom Co-Occurrence Networks Show Similar Global Topology Between Internal and External Wood

#### 3.4.1. Three Cross-Kingdom Networks Pass Stability-Based Model Selection

**Figure 3:**
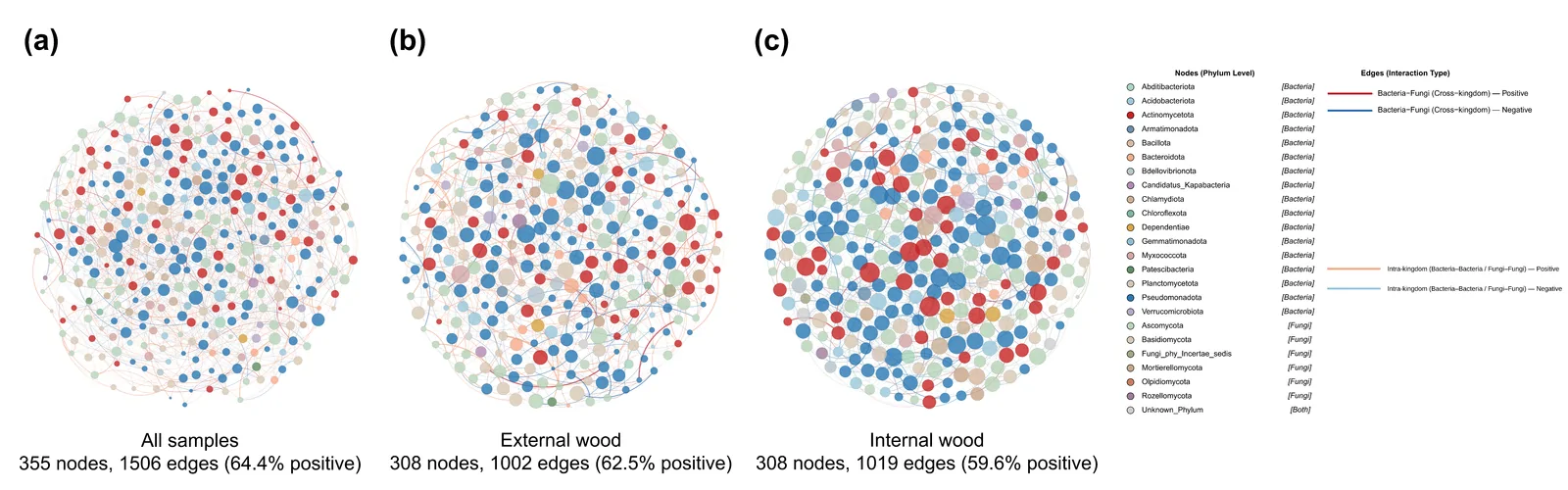
Cross-kingdom co-occurrence networks inferred with SpiecEasi (mb method, StARS selection; statistics in Table S9). (**a**) All samples (*n* = 24; 355 nodes, 1506 edges); (**b**) external wood (*n* = 12; 308 nodes, 1002 edges); (**c**) internal wood (*n* = 12; 308 nodes, 1019 edges). Nodes represent bacterial and fungal genera, coloured by phylum and sized by degree. Red and blue edges denote positive and negative associations, respectively; dark edges link different kingdoms and light edges link members of the same kingdom.

We inferred cross-kingdom association networks for all samples (*n* = 24), internal wood (*n* = 12), and external wood (*n* = 12) (Figure 3; Table S9). StARS stability values ranged from 0.0487 to 0.0496 across the three networks, and the optimal penalty parameter never fell at the boundary of the lambda path (Table S9, Section A); all three networks thus satisfied the pre-specified StARS stability criterion (≤0.05). The all-sample network comprised 355 nodes and 1506 edges; positive associations predominated in all three networks (59.6–64.4% of edges).

#### 3.4.2. Global Network Topology Does Not Differ Detectably Between Internal and External Wood

We compared the Internal and External networks—constructed as a pair on the same 308 genera—across ten global and comparative measures (number of components, average dissimilarity, average path length, density, vertex and edge connectivity, natural connectivity, percentage of positive edges, clustering coefficient, and modularity), using 500 permutations that exchange the paired samples within logs. The two networks were similar in size (1019 vs. 1002 edges; 59.6% vs. 62.5% positive edges), and no measure differed significantly after Benjamini–Hochberg adjustment (adjusted *p* = 0.20–1.00; Table S9, Section B). Vertex and edge connectivity were numerically higher in external wood (3 vs. 1; unadjusted *p* = 0.0399) but not significant after adjustment (adjusted *p* = 0.1996); we report them as descriptive observations only. Modularity was moderate in both networks (0.445 vs. 0.474; adjusted *p* = 0.353).

#### 3.4.3. Enrichment-Group Members Participate Recurrently in All Three Networks

Members of all five non-empty enrichment groups entered the all-sample network (B-EG-I 9/9; B-EG-III 21/21; F-EG-I 14/15; F-EG-III 21/21; F-EG-V 14/15; Table S9, Section C). The detection status, number of positive samples, and pooled relative abundance of every enrichment-group genus in each of the three network sample sets are reported genus by genus in Table S9, Section E. Under the shared-genus framework, most members also entered both positional networks (bacterial groups 9/9 and 21/21; fungal groups 13/15, 20/21, and 13/15 in each positional network). The mean degree of group members was broadly comparable to that of non-members, with no consistent direction of difference across networks. Cross-kingdom edges involving group members were most numerous for F-EG-III (96 edges in the all-sample network), followed by F-EG-I (68) and B-EG-III (38). Nodes classified as connectors or hubs numbered 149 (All), 128 (Internal), and 104 (External), consisting mostly of connectors (Pi > 0.62), with module hubs being rare (Table S9, Section D). Because the Zi–Pi thresholds were developed for sparse ecological networks [41], we treat these role assignments as descriptive (see Section 4.4).

### 3.5. Community–Chemistry Associations Across Decay Stages

**Figure 4:**
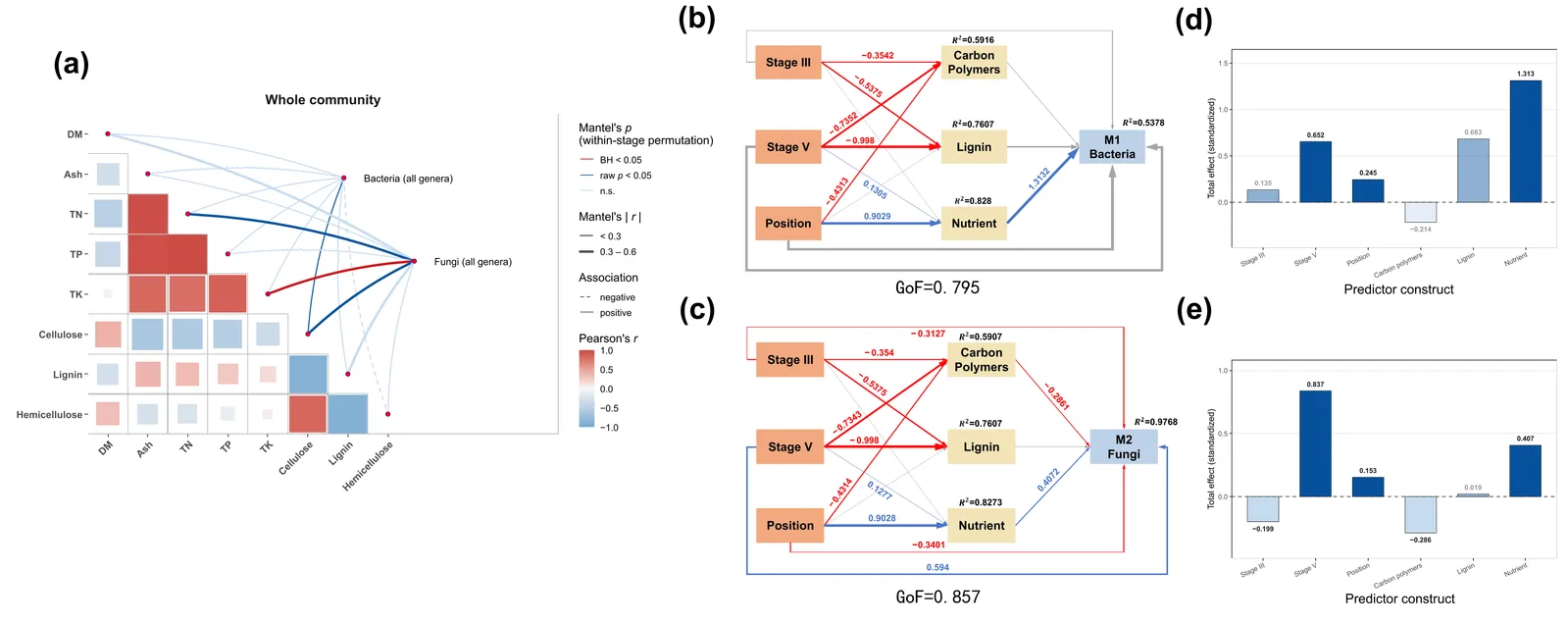
Community–chemistry associations and PLS-SEM path models (*n* = 24 samples from 12 paired logs). (**a**) Mantel associations between whole-community dissimilarity (normalized weighted UniFrac, bacteria; Bray–Curtis, fungi) and eight wood chemical properties. Lower-left triangle: Pearson correlations among properties; upper right: Mantel links, width proportional to |*r*|, colour-coded by significance under within-stage permutation (Table S10). (**b**,**c**) PLS-SEM path models for bacteria (M1) and fungi (M2). The outcome is the PCoA1 score of community composition (larger values = later decay); decay stage entered as two standardized dummies (Stage I reference). Solid arrows: bootstrap 95% CI excludes 0 (blue, positive; red, negative); dashed grey arrows: CI crosses 0; arrow width proportional to |*β*|; only significant paths are labelled. Carbon polymers = cellulose + hemicellulose; Nutrient = TN + TP + TK (g/kg fresh-wood basis). (**d**,**e**) Standardized total effects on community composition (full models); solid bars: 95% CI excludes 0. Full statistics in Table S11. In M1, the nutrient path is collinear with position (VIF = 14.8; Table S11c); the pre-registered reduced model gives *β* = +0.682 [+0.230, +1.332], the value cited in the text.

At the whole-community level, which is independent of the enrichment-group definitions, the two kingdoms differed in how closely their compositional dissimilarity tracked wood chemistry (Figure 4a; Table S10). Fungal dissimilarity showed positive coefficients with all eight chemical properties (*r* = 0.221–0.557), with the largest values for dry matter, cellulose, and lignin. Bacterial coefficients were smaller throughout (all |*r*| ≤ 0.282, largest for dry matter). Under within-stage permutations with Benjamini–Hochberg adjustment, one association remained significant, between the fungal community and total potassium $\left(r=0.348,p=0.003,p_{BH}=0.042\right)$, and the bacterial association with cellulose approached significance $\left(r=0.142,p=0.012,p_{BH}=0.098\right)$. Most associations that reached significance under free permutation of intact logs were no longer significant once permutations were restricted within decay stages (Table S10), indicating that community–chemistry associations at the whole-community level are expressed mainly along the decay-stage axis rather than the within-log positional axis. At the enrichment-group level, group dissimilarities tracked the same stage-structured chemical axes—most closely dry matter and cellulose—except for the late-decay fungal group F-EG-V, which showed no detectable association with any measured property (Table S10, Part B; Figure S5). Because group membership was defined by decay-stage enrichment, these group-level associations are expected by construction. We therefore report them in the Supplementary Materials as a characterisation of the groups.

### 3.6. PLS-SEM Reveals Contrasting Pathways Linking Decay Stage and Position to Bacterial and Fungal Communities

We used PLS-SEM to quantify how decay stage and spatial position were associated with community composition, directly and indirectly through three wood-chemistry mediators—carbon polymers (cellulose + hemicellulose), lignin, and nutrients (TN + TP + TK)—in two structurally identical models (M1, bacteria; M2, fungi; Figure 4b,c; Table S11). The decay stage was entered as two standardized dummy variables with Stage I as reference, so stage associations are interpreted jointly. The outcome was the first PCoA axis of community composition (bacteria: normalized weighted UniFrac, 89.8% of variation; fungi: Bray–Curtis, 21.8%), with larger scores corresponding to later decay. All multi-indicator blocks showed high convergent validity (loadings 0.915–0.991; AVE 0.918–0.949; composite reliability 0.971–0.974), and discriminant validity was supported by cross-loadings and HTMT ratios (Table S11a,b). Inference used cluster bootstrap resampling of the 12 logs (1999 replicates; 95% percentile CIs). Both models fitted well (GoF = 0.795 and 0.857) and explained 53.8% (adjusted $R^{2}$ = 0.375) and 97.7% (adjusted $R^{2}$ = 0.969) of variance in bacterial and fungal community composition, respectively.

Upstream paths were nearly identical between models (Figure 4b,c; Table S11d). Decay stage was negatively associated with structural carbon: Stage III and Stage V were associated with lower carbon polymers (*β* = −0.354 and −0.735 in M1; −0.354 and −0.734 in M2) and lower lignin (*β* = −0.537 and −0.998 in both models), and Stage V with slightly higher nutrients (*β* = +0.130 in M1; +0.128 in M2). Position showed the strongest upstream association: external wood was associated with markedly higher nutrient availability (*β* = +0.903 [+0.870, +0.951]) and lower carbon polymers (*β* = −0.431), but not with lignin (ns).

In the fungal model, community composition was associated with both decay stage and the measured chemistry (Figure 4c,e). Stage III and Stage V showed opposing direct associations (*β* = −0.313 [−0.479, −0.198] and +0.594 [+0.399, +0.781]), consistent with the orientation of the outcome axis. Carbon polymers were negatively (*β* = −0.286 [−0.461, −0.203]) and nutrients positively (*β* = +0.407 [+0.197, +0.936]) associated with composition, and position showed a negative direct association (*β* = −0.340 [−0.825, −0.185]). In total effects, decay stage dominated (Stage V +0.837 [+0.717, +0.935]; Stage III −0.199 [−0.320, −0.103]), whereas the total association of position was weak and positive (+0.153 [+0.025, +0.371]): its negative direct path was offset by positive indirect paths through nutrients and carbon polymers. No signal was detected along the lignin axis (total effect +0.019 [−0.132, +0.296]).

In the bacterial model, no direct path from decay stage or position to community composition was detected, and among the mediators, only nutrient availability was associated with composition (*β* = +1.313 [+0.070, +3.098]; Figure 4b,d). This coefficient exceeds unity because the nutrient axis is almost collinear with position (Position → Nutrient *β* = +0.903; VIF up to 17.6; Table S11c). The pre-registered reduced model without the lignin mediator estimated the same path at *β* = +0.682 [+0.230, +1.332], which we cite hereafter; reduced-model total effects were otherwise nearly identical to full-model estimates in both kingdoms (Table S11; Figure S6). The total associations of Stage V (+0.652 [+0.374, +0.868]) and position (+0.245 [+0.029, +0.438]) with bacterial composition were thus expressed indirectly, through the measured chemistry. Sensitivity analyses based on within-log differences (*n* = 12) and on the second PCoA axis as an alternative outcome yielded concordant patterns (Table S11f).

## 4. Discussion

### 4.1. Internal–External Chemical and Microbial Divergence Across Decay Stages

Downed log decomposition created divergent chemical microenvironments between internal and external wood, peaking at Stage III (Figure 1a). Linear mixed models with log identity as a random intercept confirmed significant Stage × Position interactions for all chemical properties after Benjamini–Hochberg adjustment ($p_{BH}$ < 0.01). Nutrient and mineral gradients between positions were largest at Stage III, whereas structural carbon fractions reversed their radial patterns across decay stages. Dry matter declined continuously, with internal wood dropping sharply from 45.80% at Stage III to 20.07% at Stage V as moisture progressively penetrated, whereas external wood decreased more gradually (31.14% to 27.23%). Total nitrogen, total phosphorus, and total potassium all peaked in external wood at Stage III (TN 10.89 g/kg, TP 0.80 g/kg, TK 2.13 g/kg) but remained low in internal wood (TN 1.84 g/kg, TP 0.10 g/kg, TK 0.56 g/kg), with TN showing a 5.9-fold disparity between positions. Total nitrogen accumulation in external wood at Stage III cannot be fully explained by internal nitrogen cycling; it may reflect exogenous inputs at the bark–wood interface, which could potentially arise from atmospheric deposition, canopy throughfall, fungal hyphal transport, or supplementary biological nitrogen fixation [51,52,53]. Lignin proportion increased internally (30.9% to 41.7%) but decreased externally (47.8% to 36.4%); cellulose and hemicellulose showed the opposite pattern. Some chemical gradients narrowed at Stage V, but the lignin gap widened again (41.74% internal vs. 36.43% external). Thus, Stage III represents the peak of nutrient and mineral heterogeneity, but late-stage divergence is reorganized rather than uniformly dampened. Cross-ecosystem evidence further indicates that deadwood microbial colonization is strongly shaped by external environmental context in addition to substrate properties [20].

Microbial alpha diversity responded asymmetrically to this gradient (Figure 1b). Bacterial Shannon diversity differed significantly by stage, position, and their interaction (Stage $p_{BH}$ = 0.028; Position $p_{BH}$ < 0.001; Stage × Position $p_{BH}$ = 0.025; Table S5). The mixed model explained 73.6% of the variance in bacterial Shannon diversity (marginal $R^{2}$ = 0.736; Table S5). External wood maintained higher bacterial diversity at Stages I and III, whereas the internal–external difference was no longer detectable at Stage V. In contrast, fungal observed ASVs showed a strong position effect across all stages (Position $p_{BH}$ < 0.001; marginal $R^{2}$ = 0.831; Table S5) and remained 2.3-fold higher in external than internal wood at Stage V (769 vs. 337), with no late-stage convergence. These patterns reflect fundamental ecological differences between the two kingdoms. Bacteria appeared to track local physicochemical gradients rapidly, and their spatial differences became undetectable as several nutrient- and moisture-related gradients narrowed by Stage V, consistent with findings that bacterial communities undergo gradual, environment-driven changes during wood decay [11]. Fungi are strongly influenced by hyphal colonization, priority effects, and substrate specialization, which anchor communities to their initial spatial niches, as experimentally demonstrated in beech wood [54]. This divergent response of bacteria and fungi to the spatial heterogeneity of downed logs is consistent with previous studies on deadwood [55].

Beta diversity revealed contrasting assembly patterns (Figure 1c). Bacterial composition did not differ significantly between Stages I and III (*p* = 0.914) or between III and V overall (*p* = 0.143); the III–V divergence was detectable only within internal wood ($R^{2}$ = 0.710, *p* = 0.029). Fungal communities differed between all stage pairs (exact *p* = 0.029), with stronger compositional shifts in internal than external wood. Because fungal multivariate dispersions also differed among stages (betadisper *p* = 0.024; Table S6), this stage effect likely combines centroid separation with differences in within-stage dispersion, and we interpret it with that qualification; bacterial dispersions were homogeneous, so the bacterial tests reflect centroid differences alone. Fungal spatial divergence intensified across stages (Stage × Position $R^{2}$ = 0.228, *p* = 0.001), with the internal–external effect size increasing from $R^{2}$ = 0.403 at Stage I to 0.563 at III and 0.812 at V. Stage III thus marks the point of strongest nutrient and mineral divergence between internal and external wood, although the spatial divergence of fungal communities continued to intensify through Stage V.

Two constraints of the design deserve note. Because wood integrity changes with decay, the sampling tool necessarily differed among stages (Section 2.2), so a sampling-method contribution to among-stage differences cannot be entirely excluded; we emphasise, however, that all internal–external contrasts were made within stages, where the tool was identical. In addition, no field negative controls or extraction blanks were included, so low-level background contamination cannot be entirely excluded for low-biomass samples.

### 4.2. Taxonomic Turnover Across Decay Stages Reflects Spatial Filtering

At Stage III, *Burkholderia-Caballeronia-Paraburkholderia* dominated internal wood (56.0%), which is consistent with previous studies [56], but it remained low externally (14.1%). This skew coincides with the strongest nutrient divergence at this stage, where internal wood had the lowest total nitrogen (1.84 g/kg) and total phosphorus (0.10 g/kg). *Pseudomonas* showed a similar early-internal preference (14.6% at Stage I) but declined thereafter. Other abundant genera, including *Sphingomonas* and unclassified Micropepsaceae, maintained lower background abundances.

Fungal communities followed a directional path. Ascomycota dominated early decay (77.2% external, 60.9% internal at Stage I). Basidiomycota then progressively replaced Ascomycota (78.1% internal, 52.3% external at Stage III; 68.1–73.0% at Stage V). Internal wood showed earlier Basidiomycota dominance, suggesting faster internal substrate transformation. This shift represents a compositional turnover toward wood-decay specialists. Together, these patterns reinforce the framework from Section 4.1: bacteria act as rapid responders to local filters, while fungi follow decay-class-associated paths.

### 4.3. Differentially Abundant Taxa Form Stage- and Position-Associated Enrichment Groups

The differentially abundant taxa identified by ALDEx2 clustered into distinct stage- and position-associated enrichment groups (Section 3.3.3; Table S8), and we organize this discussion around these groups. We stress that the groups are empirical, enrichment-based taxonomic groupings: their functional interpretation is hypothesised from taxonomy, FUNGuild and FAPROTAX predictions, and the literature rather than measured in this system, and validating such roles will require metatranscriptomic or enzymatic evidence.

Bacterial enrichment was confined to internal wood and to the earlier decay stages. B-EG-I taxa were enriched in Stage I internal wood, where *Pseudomonas* reached 14.6% relative abundance. B-EG-III taxa peaked in Stage III internal wood—dominated by the *Burkholderia-Caballeronia-Paraburkholderia* complex (56.0%) alongside *Luteibacter*, *Gryllotalpicola*, *Acidisoma*, and *Nocardioides*—coinciding with the strongest nutrient and mineral divergence between positions (internal TN = 1.84 g/kg, TP = 0.10 g/kg; Section 4.1). No bacterial genus was enriched toward Stage V, no bacterial genus showed significant position-associated enrichment, and no significant genus was detected in external wood: bacterial differentiation in these logs was a mid-decay, internal-wood phenomenon.

Fungal enrichment, in contrast, spanned the entire decay sequence and was mainly directed toward the log interior. F-EG-I taxa (e.g., *Botryobasidium*, *Armillaria*, *Scytalidium*) were enriched in early internal decay. F-EG-III taxa (e.g., *Resinicium*, *Lentinellus*, *Mycena*) peaked at Stage III, coinciding with the same nutrient-depleted internal microenvironment that marked the bacterial peak. F-EG-V taxa (e.g., *Lasiosphaeris*, *Stropharia*, *Phallus*, *Dacryopinax*, *Tricholomopsis*) were enriched in Stage V internal wood, and every fungal genus with significant position-associated enrichment at all three decay stages was enriched internally. Late-decay replacement of differentially abundant taxa was thus carried entirely by fungi, in contrast to the empty bacterial B-EG-V.

Within these empirical groups, several genera have documented capabilities that are consistent with their enrichment positions, although we emphasise that these roles are hypothesised from functional prediction and the literature rather than measured in this system (Table S13). FAPROTAX resolved most bacterial members only to aerobic chemoheterotrophy, the default assignment for these lineages; the one more specific prediction was aromatic-compound degradation in *Nocardioides* (B-EG-III). The literature adds further, still hypothesised, capabilities. Among the early-decay groups, *Penicillium* is a reported pioneer coloniser of woody substrates under nutrient-poor conditions [57], and lignin-degrading *Pseudomonas* isolates have been recovered from decaying wood [58]. The mid-decay groups, enriched where internal nutrients were scarcest, contain genera with polysaccharide-depolymerising potential: *Luteibacter* carries lignocellulose-degrading gene repertoires [59], *Trichoderma* combines cellulolytic activity with mycoparasitism on other wood-inhabiting fungi [60], and *Mycena* includes late-phase degraders of already softened wood [61]. The late-decay group F-EG-V includes *Podospora*, reported to use the more recalcitrant lignocellulose fraction as a late-successional degrader [62], the wood-associated *Lasiosphaeris* [63], and the wood saprotroph *Dacryopinax*. Consistent with this pattern, FUNGuild assigned nearly all enrichment-group fungi to saprotrophic guilds, mostly at Probable confidence or higher; the single exception was *Chloridium*, assigned as ectomycorrhizal at only Possible confidence (Table S13). These hypothesised roles describe a stage-ordered functional potential that parallels the chemical gradient; whether members of these groups also associate with one another is examined by the network analysis (Section 4.4).

The two kingdoms therefore exhibited complementary enrichment patterns along the decay gradient. Bacterial enrichment was concentrated where and when nutrient and mineral heterogeneity peaked, whereas fungal enrichment extended through late decay and into the most chemically altered internal microhabitats. These patterns are consistent with a decay-class-associated sequence in which bacterial taxa respond to the mid-decay internal chemical gradient and fungal taxa predominate in late-decay internal wood. The participation of these groups in cross-kingdom association networks is examined in Section 3.4 and discussed in Section 4.4.

### 4.4. Cross-Kingdom Association Networks Show a Shared Structure Across Positions

Three properties of the inferred networks deserve emphasis. First, all three networks satisfied the pre-specified StARS stability criterion (≤0.05; Table S9). Given that even the largest sample sets comprise 12 observations against 308 candidate genera, we treat these networks as exploratory association analyses: they satisfy their stated quality criterion, but we place no further weight on individual edges. Second, positive associations predominated in every network (59.6–64.4% of edges). Third, we stress that these edges are statistical associations: co-occurrence is not evidence of ecological interaction [24], and we interpret the networks accordingly throughout.

The marked chemical and compositional divergence between internal and external wood (Section 3.1 and Section 4.1) was not mirrored by network topology: none of the ten global and comparative measures differed significantly between the two positional networks after multiple-testing correction (Table S9, Section B). We regard this as a statistically grounded negative result within the limits of twelve paired logs, not as evidence that the two networks are identical. Vertex and edge connectivity were numerically higher in external wood, but the difference did not survive correction, and we do not interpret it further. Modularity was moderate (0.445–0.474), similar between positions, and is reported as a descriptive statistic only.

The enrichment groups defined by differential abundance were recurrent participants of the association structure: members of all five non-empty groups entered the all-sample network, and most also entered both positional networks (Table S9, Section C). Members of F-EG-III—the group enriched at the mid-decay peak of internal–external chemical heterogeneity—were involved in the largest number of cross-kingdom edges (96 in the all-sample network), followed by F-EG-I (68) and B-EG-III (38). At the same time, their mean degrees were unremarkable relative to non-members, so the present evidence does not single them out as network coordinators.

Connector and hub assignments followed the same pattern of restraint. Connectors (Pi > 0.62) were numerous in all three networks (104–149 nodes), whereas module hubs were rare (Table S9, Section D). Because the Zi–Pi thresholds were developed for sparse pollination networks [41] and are easily exceeded in denser microbial networks such as ours, we report these roles as descriptive features and refrain from functional interpretation of individual connectors.

Read together with Section 4.1, Section 4.2 and Section 4.3, the network results show that the two kingdoms, despite differing strongly in composition and enrichment patterns, are embedded in association networks that are topologically alike between positions. What these patterns mean mechanistically remains open: the present data cannot distinguish whether the divergent responses of the two kingdoms arise from shared responses to the same chemical environment, from direct or indirect cross-kingdom interactions, or from a combination of both [22,24]. Resolving this will require process-level evidence such as enzyme activities or metatranscriptomic measurements.

### 4.5. Contrasting Community–Chemistry Associations of Bacteria and Fungi

The Mantel results add a distance-based layer to the contrasting responses of the two kingdoms (Section 4.1). Fungal community dissimilarity showed positive associations with all eight measured chemical properties, whereas bacterial coefficients were smaller throughout and largest for dry matter. This asymmetry parallels the beta-diversity patterns, in which fungal composition was structured more strongly by decay stage and position than bacterial composition (Section 3.1), and it is consistent with broad chemical tracking by fungi reported at larger spatial scales [64]. Bacterial associations were weaker but not absent: beyond dry matter, the bacterial community showed a trend-level association with cellulose, a pattern compatible with bacteria responding to several concurrent filters rather than to any single chemical axis.

One interpretive boundary deserves emphasis. Because the enrichment groups were defined by decay-stage enrichment, their associations with stage-structured chemistry are expected by construction, and we do not present them as independent evidence of environmental filtering. The within-stage permutation scheme quantifies how much association remains once the decay-stage axis is held fixed: of the 56 community- and group-level tests, 33 reached significance under nominal free permutation of intact logs, but only one—that between the fungal community and total potassium—remained significant under within-stage permutation after Benjamini–Hochberg adjustment (Table S10). Community–chemistry associations in these logs are therefore expressed predominantly along the decay-stage axis, and residual associations beyond that axis are rare.

The late-decay fungal group F-EG-V departs from this stage-scale pattern: it showed no detectable association with any measured chemical property under any permutation scheme (Table S10, Part B). One possible explanation is that late-decay fungal specialists, once established, become anchored by hyphal networks and priority effects and thus less sensitive to the local chemical environment [25,54]. We offer this as a hypothesis; distinguishing such anchoring from simple insensitivity to the variables we measured will require process-level evidence.

Mantel tests are symmetric and carry no directionality: they establish that community composition and chemistry co-vary, not which side of the association responds to the other. To quantify directional associations along a prespecified path structure—from decay stage and spatial position to community composition through chemical mediators—we turn to path modelling in Section 4.6.

### 4.6. Decay Stage and Position Relate to the Two Kingdoms Through Contrasting Pathways

The path models evaluate directional associations within a prespecified path structure, complementing the symmetric, distance-based patterns of Section 4.5, and they sharpen the contrast between the two kingdoms. Fungal community composition was associated primarily with the decay-stage axis: the total associations of Stage III and Stage V (−0.199 and +0.837) mirror the ordering of fungal PCoA1 scores across stages (group means −0.134, −0.260, and +0.394 at Stages I, III, and V; correlation with stage order *r* = 0.62) and agree with the stage-structured PERMANOVA and enrichment patterns reported above. Because the outcome axis is itself strongly stage-structured and is spanned by two stage dummies, the high $R^{2}$ of the fungal model (0.977) reflects this property of the outcome axis. In the fungal model, wood chemistry occupied an intermediate position in the prespecified path structure: carbon depletion (carbon polymers, −0.286) and nutrient enrichment (+0.407) were each associated with composition independently of stage.

Position presented a suppression pattern in the fungal model. Its direct association was negative (*β* = −0.340), but its indirect associations through nutrients (+0.903 × +0.407 ≈ +0.37) and carbon polymers (−0.431 × −0.286 ≈ +0.12) were positive, so the net total association was only weakly positive (+0.153). We interpret this as two opposing channels: position was associated with fungal composition negatively through routes not represented by the measured chemistry—plausibly unmeasured microenvironmental filters such as oxygen availability, moisture, and physical barriers—and positively through the measured chemical template.

Bacterial composition showed the opposite architecture: no direct association with stage or position, and a single detectable channel through nutrients (reduced-model *β* = +0.682). This agrees with the weaker bacterial community–chemistry associations in the Mantel analysis (Section 4.5) and with the modest position effect in PERMANOVA ($R^{2}$ = 0.071, *p* = 0.038; Section 3.1). Two qualifications apply to all non-significant paths. First, with 12 independent logs, statistical power is limited. Second, the mediators covered only eight bulk chemical properties: unmeasured factors (oxygen, moisture, pH, competition) may carry associations that our model cannot represent, so a non-significant path means that no signal was detected along the measured axes—not that the axis is ecologically irrelevant.

One construct deserves explicit caution. No independent signal was detected along the lignin axis in either kingdom, but because lignin was entangled with the stage and position gradients (VIF = 10.3; Table S11c), its coefficient is not separately identifiable; we therefore refrain from interpreting it in either direction. Overall, the two models converge on a tiered picture: decay stage structured both the chemical template and the fungal community, whereas position structured the nutrient axis and—mainly through it—bacterial composition. This pathway-level contrast reinforces the conclusion of Section 4.4: the two kingdoms responded differently to the same within-log gradients, and our design cannot distinguish whether these divergent patterns reflect shared environmental responses, direct or indirect cross-kingdom interactions, or a combination of both at the scales and time points we sampled.

### 4.7. Limitations and Future Directions

Several limitations bound the scope of these findings. We studied a single tree species at one site, sampled on one occasion with decay stage as a space-for-time substitute, so the patterns reported here are most directly applicable to temperate coniferous forests and should be tested across other tree species and climatic zones before wider extrapolation. With twelve paired logs, statistical power is limited—particularly for the network comparisons and the path models—and we therefore read non-significant results as “not detected” rather than “absent” (Section 4.4 and Section 4.6). The path models also covered only eight bulk chemical properties: unmeasured microenvironmental factors (oxygen, moisture, pH, physical barriers, and competition) may carry associations our design cannot capture, no process-level measurements (mass loss, enzyme activities, or gene expression) were taken, and co-occurrence edges remain statistical associations rather than demonstrated ecological interactions [24]. Likewise, the functions attributed to enrichment groups are hypothesised from the literature, FAPROTAX, and FUNGuild evidence of varying confidence (Table S13) and await metatranscriptomic or enzymatic verification; the stage-dependent change of sampling tool and the absence of field blanks are acknowledged in Section 4.1. Future work should combine metatranscriptomic and enzyme-activity measurements with microenvironmental profiling in time-series sampling across tree species and climate gradients.

## 5. Conclusions

In *Larix principis-rupprechtii* downed logs, internal and external wood differed in both chemistry and microbial community composition, and this internal–external chemical heterogeneity peaked at the intermediate decay stage (Stage III; a 5.9-fold difference in total nitrogen). The two kingdoms responded to this spatial template in contrasting ways: bacterial diversity converged between positions by late decay, whereas fungal communities maintained persistent and deepening internal–external divergence across all stages. Our path models showed that decay stage and position were associated with the two kingdoms through different pathways—fungal composition tracked primarily the decay-stage axis (Stage V total association +0.837), whereas bacterial composition was associated more weakly and mainly through an indirect nutrient channel +0.682. These patterns are most directly applicable to temperate coniferous forests; testing them across tree species, climate gradients, and time-series sampling with process-level measurements is the natural next step.

## Data Availability

The raw 16S rRNA (V5–V7) and ITS1 amplicon sequencing data have been deposited in the NCBI Sequence Read Archive under BioProject accession PRJNA1499194 (https://www.ncbi.nlm.nih.gov/bioproject/PRJNA1499194, accessed on 21 August 2026). The wood physicochemical properties of all samples are provided in Table S1 of the Supplementary Materials. The R analysis scripts (run in R version 4.5.3) are openly available on GitHub at https://github.com/KevinChenZH/Downed-Logs-Microbiome-Analysis-Code (accessed on 21 August 2026). Per-sample BioSample and SRA run accessions are provided in Supplementary Table S12.

## Supplementary Materials

The following supporting information can be downloaded at: https://www.mdpi.com/article/10.3390/microorganisms14091885/s1, Table S1: Raw physicochemical properties; Table S2: Linear mixed model results for wood physicochemical properties; Table S3: Alpha diversity values and post-hoc contrasts; Table S4: Alpha diversity LMM diagnostics; Table S5: Alpha diversity LMM omnibus tests; Table S6: PERMANOVA and multivariate dispersion results; Table S7: ALDEx2 differential abundance summary across 18 pairwise comparisons; Table S8: Enrichment group assignments; Table S9: Cross-kingdom network statistics; Table S10: Mantel tests (Part A: whole community; Part B: enrichment groups); Table S11: PLS-SEM measurement, validity, collinearity, path, effect, and sensitivity statistics; Table S12: BioSample and SRA run accessions; Table S13: FAPROTAX-FUNGuild functional assignments; Figure S1: Linear mixed model $R^{2}$ for wood physicochemical properties; Figure S2: Temporal and spatial dynamics of community composition; Figure S3: MA plots for the four featured differential abundance comparisons; Figure S4: Volcano plots for the twelve comparisons; Figure S5: Enrichment-group Mantel associations with wood chemistry; Figure S6: PLS-SEM total effects in the pre-registered reduced models; Figure S7: PLS-SEM direct, indirect, and total effect decomposition.
