# Supplementary material for: Internal–External Heterogeneity in Downed Logs Across Decay Stages: Divergent Wood Chemistry and Contrasting Bacterial and Fungal Responses
Source: Microorganisms. 2026 Aug 25;14(9):1885. doi: 10.3390/microorganisms14091885 (PMC13609650; doi:10.3390/microorganisms14091885)
Supplement: Supplementary file 1 [file microorganisms-14-01885-s001.zip › Figure_S1_Linear mixed model R2 for wood physicochemical properties.pdf]

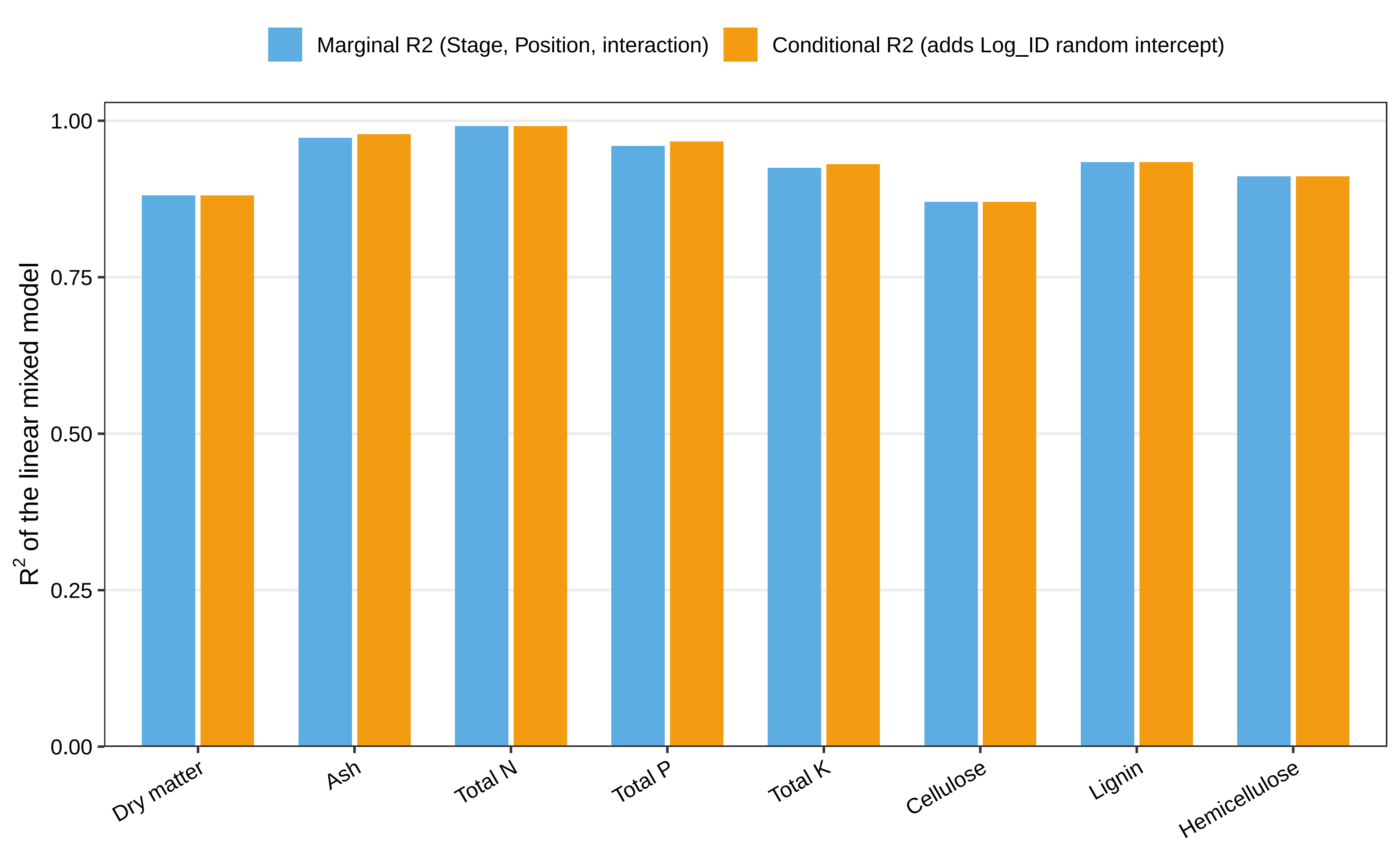

Marginal R2 (Stage, Position, interaction)
Conditional R2 (adds Log_ID random intercept)
R^2 of the linear mixed model
1.00
0.75
0.50
0.25
0.00
Dry matter
Ash
Total N
Total P
Total K
Cellulose
Lignin
Hemicellulose
