# Supplementary material for: Internal–External Heterogeneity in Downed Logs Across Decay Stages: Divergent Wood Chemistry and Contrasting Bacterial and Fungal Responses
Source: Microorganisms. 2026 Aug 25;14(9):1885. doi: 10.3390/microorganisms14091885 (PMC13609650; doi:10.3390/microorganisms14091885)
Supplement: Supplementary file 1 [file microorganisms-14-01885-s001.zip › Figure_S2_Temporal and spatial dynamics of community composition.pdf]

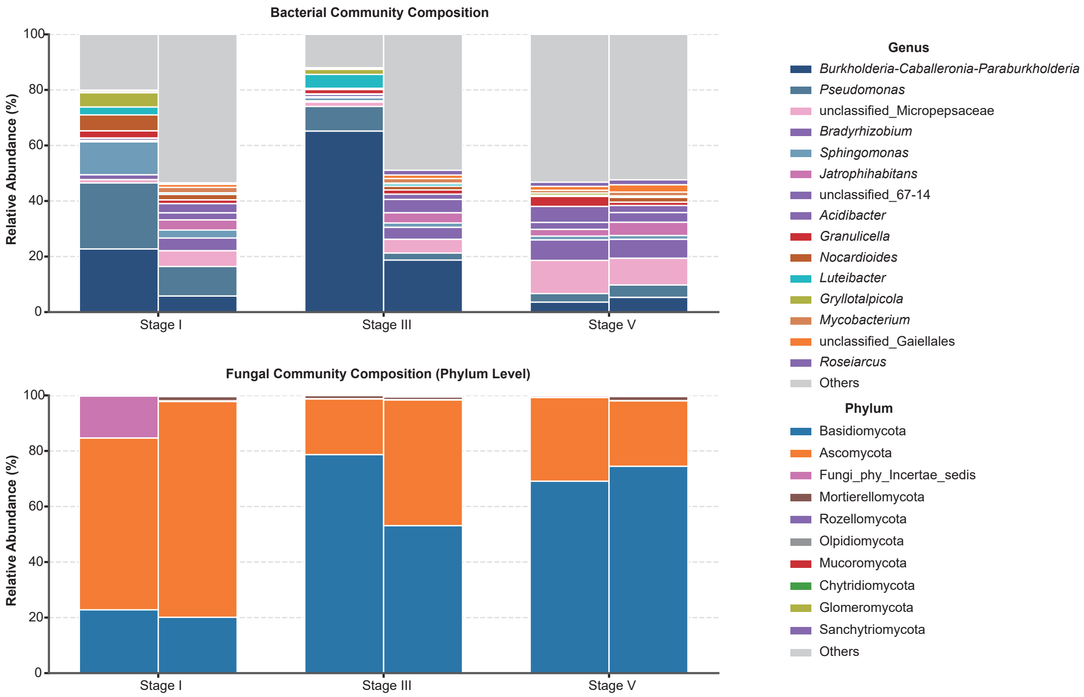

Bacterial Community Composition
Relative Abundance (%)
0
20
40
60
80
100
Stage I
Stage III
Stage V
Fungal Community Composition (Phylum Level)
Relative Abundance (%)
0
20
40
60
80
100
Stage I
Stage III
Stage V
Genus
Burkholderia-Caballeronia-Paraburkholderia
Pseudomonas
unclassified_Micropepsaceae
Bradyrhizobium
Sphingomonas
Jatrophihabitans
unclassified_67-14
Acidibacter
Granulicella
Nocardioides
Luteibacter
Gryllotalpicola
Mycobacterium
unclassified_Gaiellales
Roseiarcus
Others
Phylum
Basidiomycota
Ascomycota
Fungi_phy_Incertae_sedis
Mortierellomycota
Rozellomycota
Olpidiomycota
Mucoromycota
Chytridiomycota
Glomeromycota
Sanchytriomycota
Others
