# Supplementary material for: Internal–External Heterogeneity in Downed Logs Across Decay Stages: Divergent Wood Chemistry and Contrasting Bacterial and Fungal Responses
Source: Microorganisms. 2026 Aug 25;14(9):1885. doi: 10.3390/microorganisms14091885 (PMC13609650; doi:10.3390/microorganisms14091885)
Supplement: Supplementary file 1 [file microorganisms-14-01885-s001.zip › Figure_S3_MA plots for the four featured differential abundance comparisons.pdf]

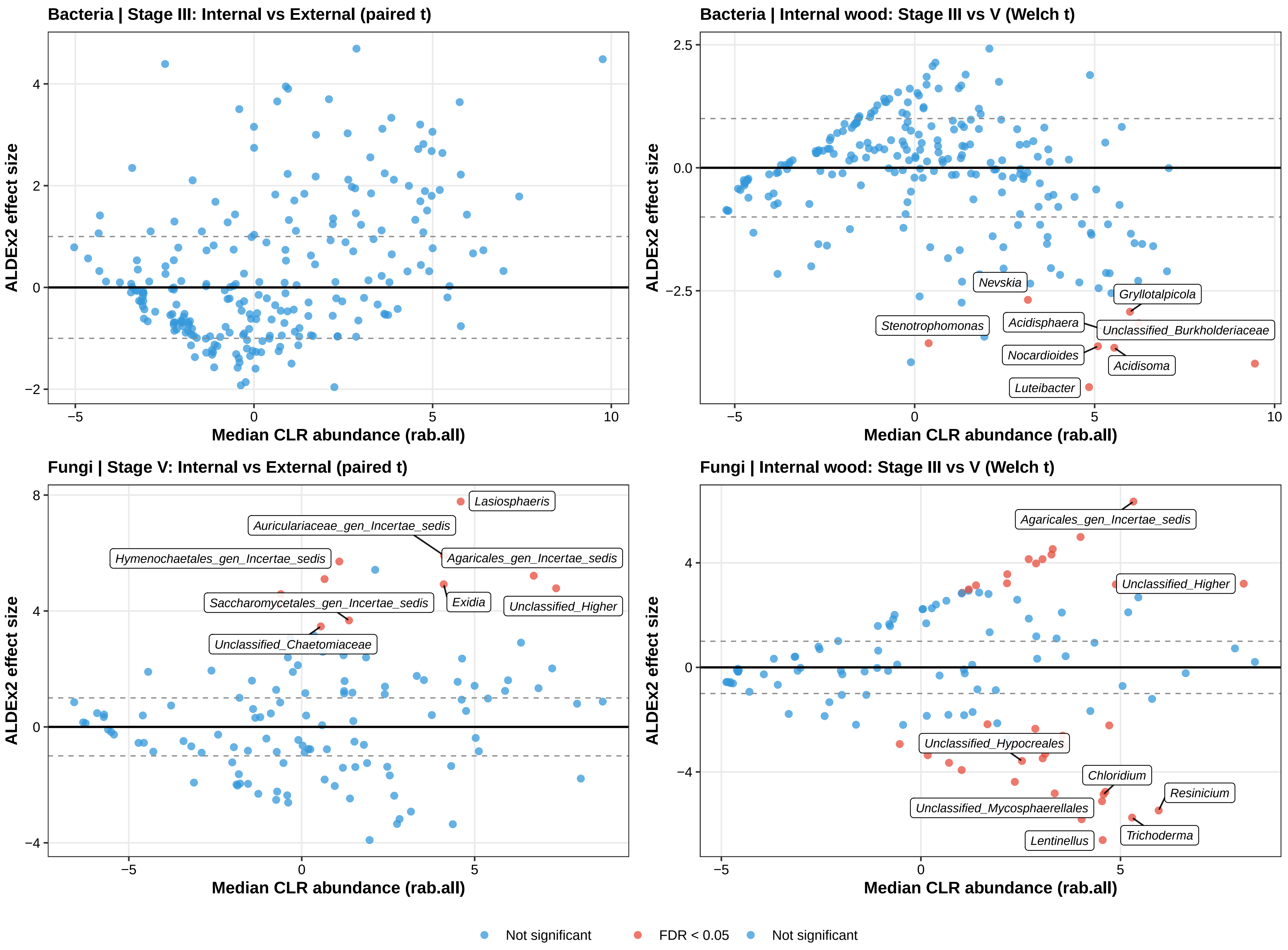

Bacteria | Stage III: Internal vs External (paired t)
Bacteria | Internal wood: Stage III vs V (Welch t)
Fungi | Stage V: Internal vs External (paired t)
Fungi | Internal wood: Stage III vs V (Welch t)
ALDEx2 effect size
Median CLR abundance (rab.all)
Nevskia
Gryllotalpicola
Stenotrophomonas
Acidisphaera
Unclassified_Burkholderiaceae
Nocardioides
Acidisoma
Luteibacter
Lasiosphaeris
Auriculariaceae_gen_Incertae_sedis
Hymenochaetales_gen_Incertae_sedis
Agaricales_gen_Incertae_sedis
Saccharomycetales_gen_Incertae_sedis
Exidia
Unclassified_Higher
Unclassified_Chaetomiaceae
Unclassified_Hypocreales
Chloridium
Resinicium
Unclassified_Mycosphaerellales
Trichoderma
Lentinellus
Not significant
FDR < 0.05
Not significant
