# Supplementary material for: Internal–External Heterogeneity in Downed Logs Across Decay Stages: Divergent Wood Chemistry and Contrasting Bacterial and Fungal Responses
Source: Microorganisms. 2026 Aug 25;14(9):1885. doi: 10.3390/microorganisms14091885 (PMC13609650; doi:10.3390/microorganisms14091885)
Supplement: Supplementary file 1 [file microorganisms-14-01885-s001.zip › Figure_S4_Volcano plots for the twelve comparisons.pdf]

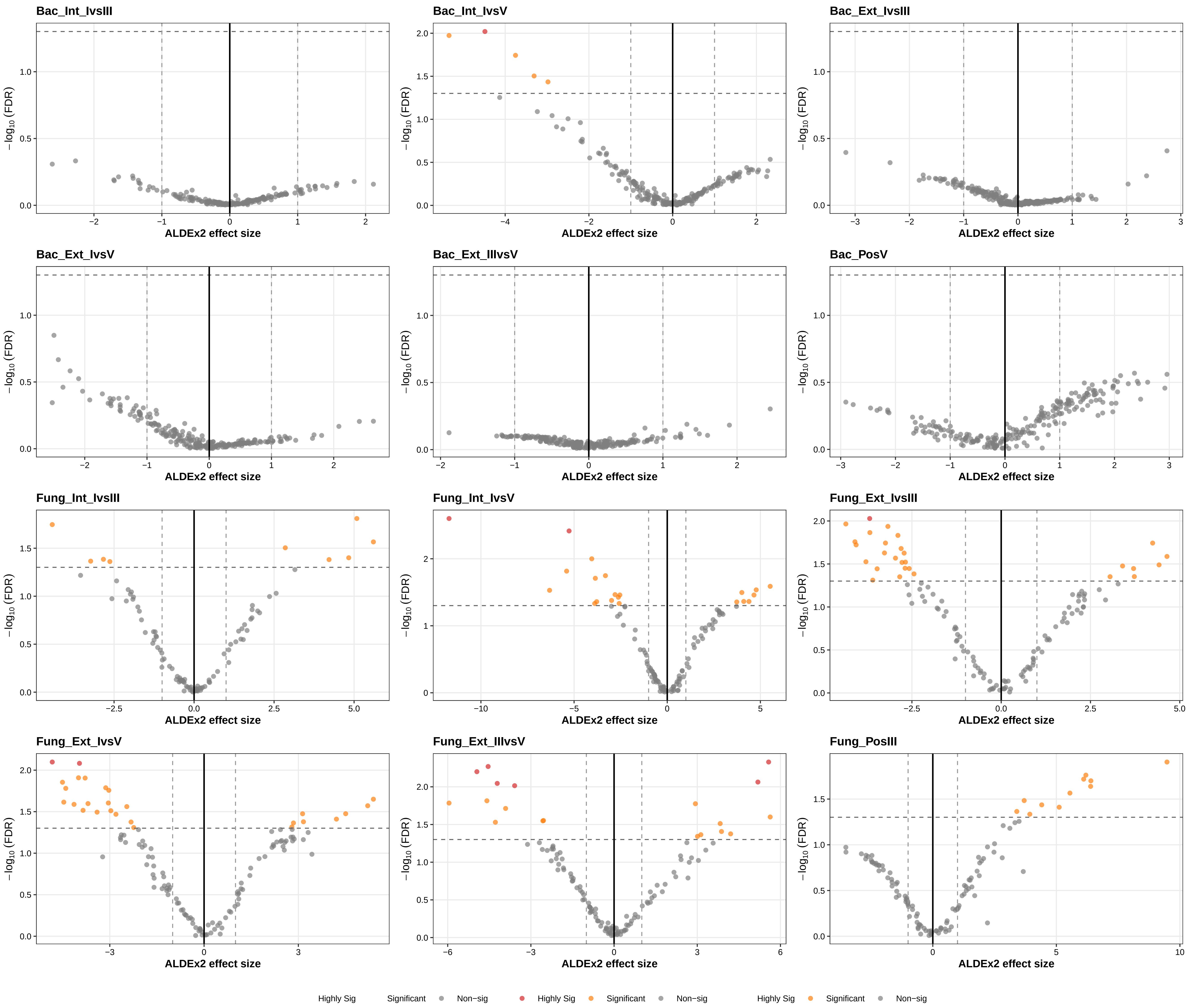

Bac_Int_IvsIII
Bac_Int_IvsV
Bac_Ext_IvsIII
Bac_Ext_IvsV
Bac_Ext_IIIvsV
Bac_PosV
Fung_Int_IvsIII
Fung_Int_IvsV
Fung_Ext_IvsIII
Fung_Ext_IvsV
Fung_Ext_IIIvsV
Fung_PosIII
$-\log_{10}$(FDR)
ALDEx2 effect size
Highly Sig
Significant
Non−sig
Highly Sig
Significant
Non−sig
Highly Sig
Significant
Non−sig
