# Supplementary material for: Internal–External Heterogeneity in Downed Logs Across Decay Stages: Divergent Wood Chemistry and Contrasting Bacterial and Fungal Responses
Source: Microorganisms. 2026 Aug 25;14(9):1885. doi: 10.3390/microorganisms14091885 (PMC13609650; doi:10.3390/microorganisms14091885)
Supplement: Supplementary file 1 [file microorganisms-14-01885-s001.zip › Figure_S5_Enrichment-group Mantel associations with wood chemistry.pdf]

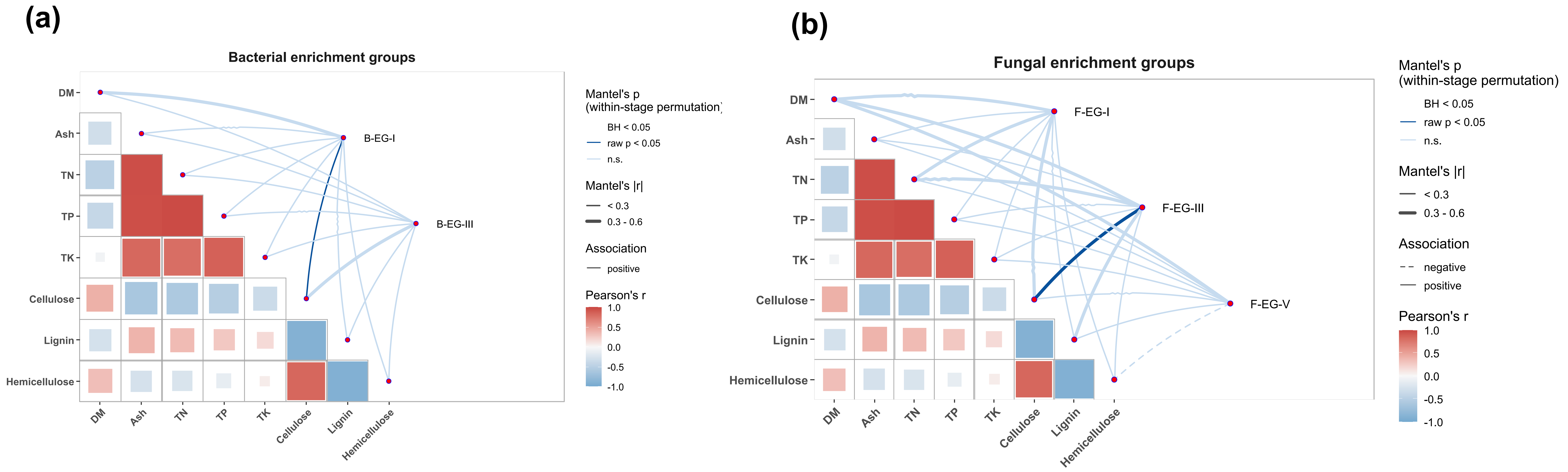
(a)
Bacterial enrichment groups
DM
Ash
TN
TP
TK
Cellulose
Lignin
Hemicellulose
B-EG-I
B-EG-III
Mantel's p
(within-stage permutation)
BH < 0.05
raw p < 0.05
n.s.
Mantel's |r|
< 0.3
0.3 - 0.6
Association
positive
Pearson's r
1.0
0.5
0.0
-0.5
-1.0
(b)
Fungal enrichment groups
DM
Ash
TN
TP
TK
Cellulose
Lignin
Hemicellulose
F-EG-I
F-EG-III
F-EG-V
Mantel's p
(within-stage permutation)
BH < 0.05
raw p < 0.05
n.s.
Mantel's |r|
< 0.3
0.3 - 0.6
Association
negative
positive
Pearson's r
1.0
0.5
0.0
-0.5
-1.0
