# Supplementary material for: Internal–External Heterogeneity in Downed Logs Across Decay Stages: Divergent Wood Chemistry and Contrasting Bacterial and Fungal Responses
Source: Microorganisms. 2026 Aug 25;14(9):1885. doi: 10.3390/microorganisms14091885 (PMC13609650; doi:10.3390/microorganisms14091885)
Supplement: Supplementary file 1 [file microorganisms-14-01885-s001.zip › Figure_S6_PLS-SEM total effects in the pre-registered reduced models.pdf]

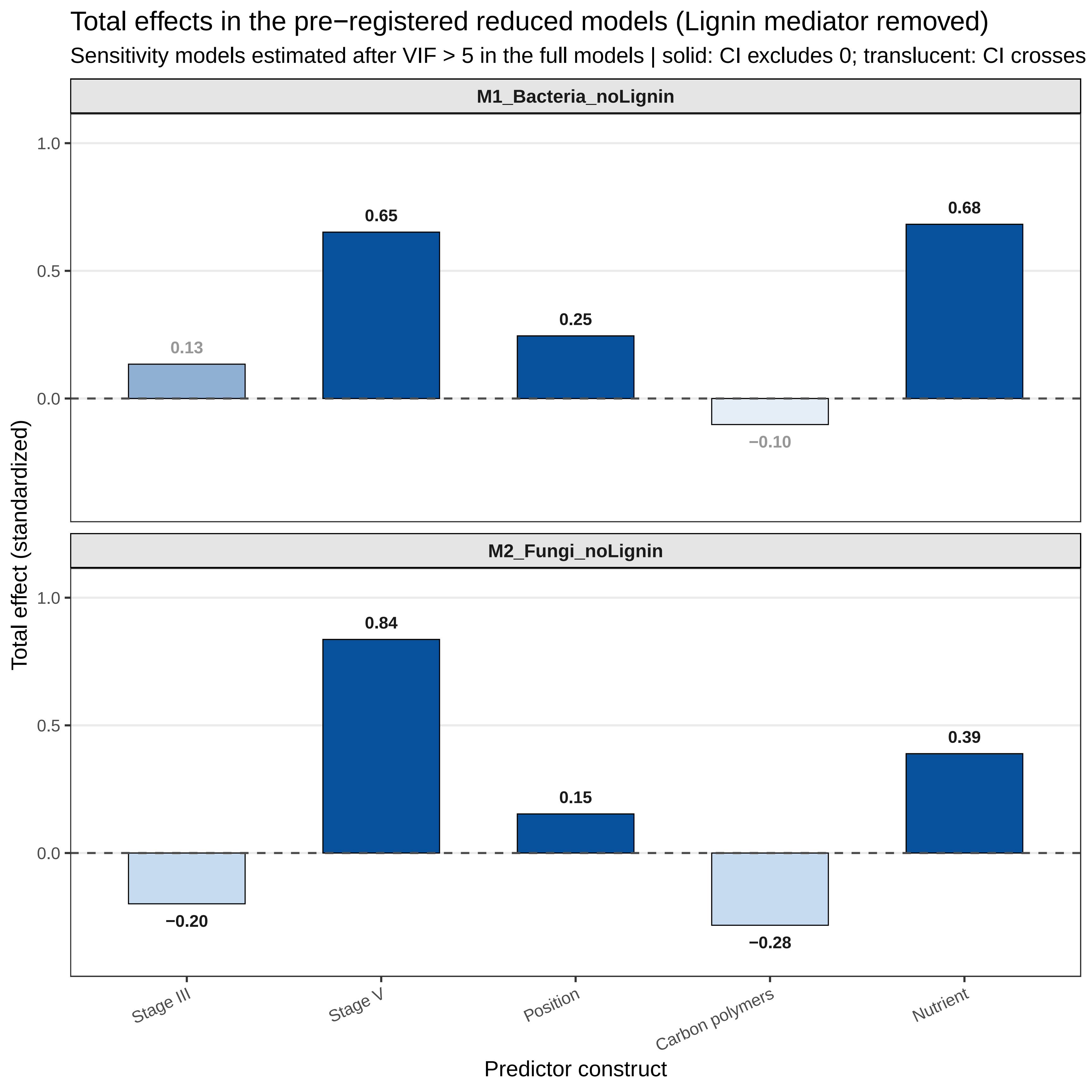
Total effects in the pre−registered reduced models (Lignin mediator removed)
Sensitivity models estimated after VIF > 5 in the full models | solid: CI excludes 0; translucent: CI crosses
M1_Bacteria_noLignin
M2_Fungi_noLignin
Total effect (standardized)
1.0
0.5
0.0
0.13
0.65
0.25
−0.10
0.68
0.84
0.15
0.39
−0.20
−0.28
Stage III
Stage V
Position
Carbon polymers
Nutrient
Predictor construct
