# Supplementary material for: Internal–External Heterogeneity in Downed Logs Across Decay Stages: Divergent Wood Chemistry and Contrasting Bacterial and Fungal Responses
Source: Microorganisms. 2026 Aug 25;14(9):1885. doi: 10.3390/microorganisms14091885 (PMC13609650; doi:10.3390/microorganisms14091885)
Supplement: Supplementary file 1 [file microorganisms-14-01885-s001.zip › Figure_S7_PLS-SEM direct, indirect, and total effect decomposition.pdf]

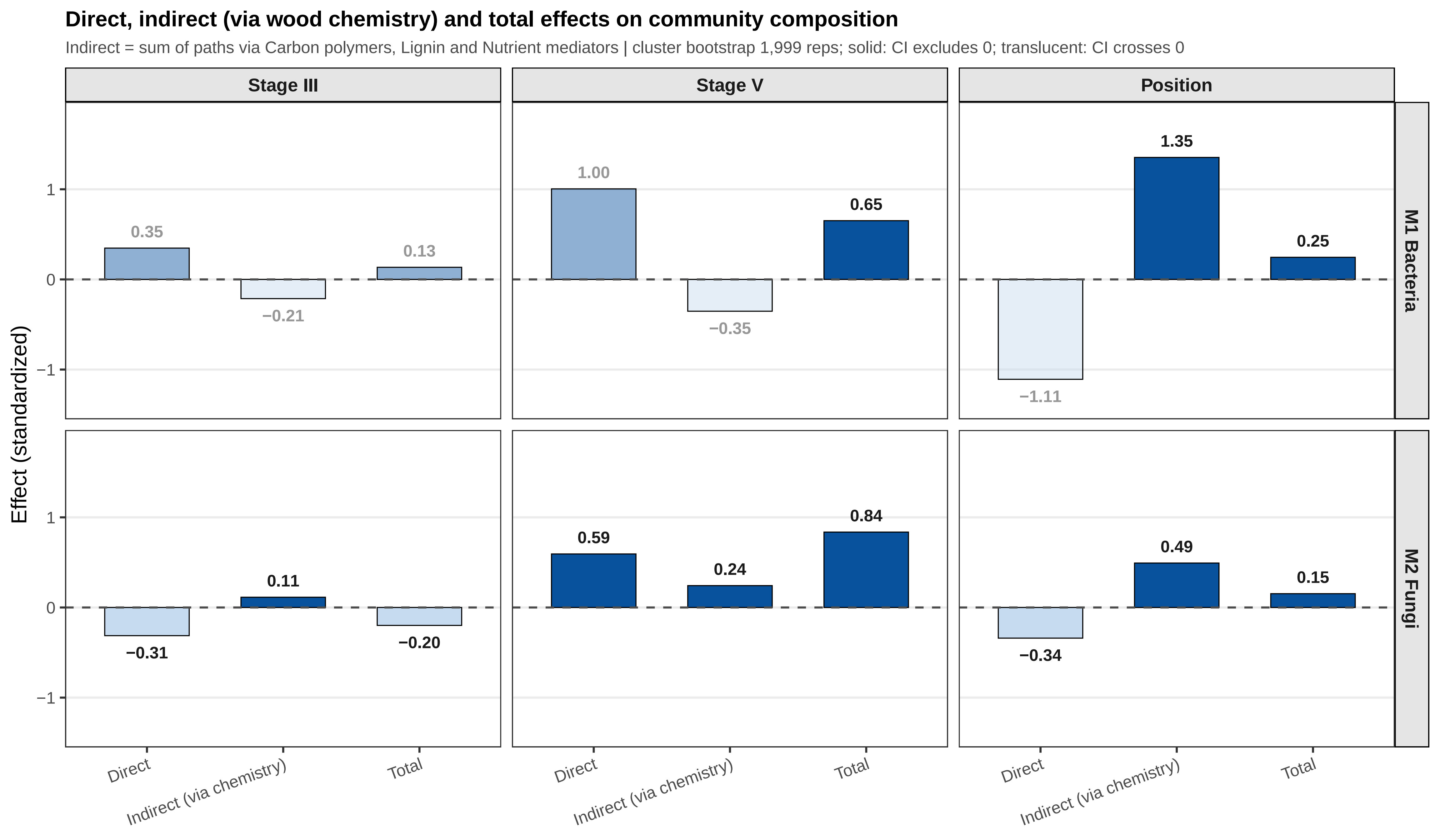

Direct, indirect (via wood chemistry) and total effects on community composition
Indirect = sum of paths via Carbon polymers, Lignin and Nutrient mediators | cluster bootstrap 1,999 reps; solid: CI excludes 0; translucent: CI crosses 0
Stage III
Stage V
Position
M1 Bacteria
M2 Fungi
Effect (standardized)
1
0
−1
0.35
−0.21
0.13
1.00
−0.35
0.65
−1.11
1.35
0.25
−0.31
0.11
−0.20
0.59
0.24
0.84
−0.34
0.49
0.15
Direct
Indirect (via chemistry)
Total
