# Supplementary material for: Internal–External Heterogeneity in Downed Logs Across Decay Stages: Divergent Wood Chemistry and Contrasting Bacterial and Fungal Responses
Source: Microorganisms. 2026 Aug 25;14(9):1885. doi: 10.3390/microorganisms14091885 (PMC13609650; doi:10.3390/microorganisms14091885)
Supplement: Supplementary file 1 [file microorganisms-14-01885-s001.zip › Supporting Information.pdf]

Supporting Information

Internal–External Heterogeneity in Downed Logs Across Decay Stages: Divergent Wood Chemistry and Contrasting Bacterial and Fungal Responses

Zhihao Chen, Yiqiong Zhang, Zhouchang Zhang, Tianjiao Song, Yufei Ji, Xinrui Li,

Liangxuan Xu, Hua Yi, Yao Wang and Yanbing Lin[*]

College of Life Sciences, Northwest A&F University, Yangling 712100, China; chenzhihao@nwafu.edu.cn (Z.C.); zhangyq0099@nwafu.edu.cn (Y.Z.); zhouchangzhang@nwafu.edu.cn (Z.Z.); 2023060644@nwafu.edu.cn (T.S.); jiyufei@nwafu.edu.cn (Y.J.); xinrui-li@nwafu.edu.cn (X.L.); xlx1111@nwafu.edu.cn (L.X.); yihua0406@nwafu.edu.cn (H.Y.); wangyao@nwsuaf.edu.cn (Y.W.)

* Correspondence: linyb2004@nwafu.edu.cn

Tables

Table S1. Raw physicochemical properties of the 24 downed-log samples: dry matter, ash, total nitrogen (TN), total phosphorus (TP), total potassium (TK), cellulose, hemicellulose, and lignin, together with sample identities (SampleID, Log_ID, decay Stage, Position, and Stage × Position Group).

Table S2. Linear mixed model results for wood physicochemical properties: omnibus Type III tests (Stage, Position, Stage × Position) for the eight properties, fitted as value ~ Stage × Position $+(1|Log_I D)$ by REML with Satterthwaite denominator degrees of freedom and Benjamini–Hochberg adjustment; models with boundary (zero random-intercept variance) fits are flagged.

Table S3. Alpha diversity values and post-hoc contrasts: per-sample alpha diversity indices (Chao1 richness, observed ASVs, Shannon index, Pielou's evenness) for both kingdoms, and planned Internal-vs-External contrasts within each decay stage from estimated marginal means.

Table S4. Alpha diversity LMM diagnostics: model diagnostics for the alpha-diversity linear mixed models, including DHARMa residual checks and singular (boundary) fit reporting.

Table S5. Alpha diversity LMM omnibus tests: Type III tests (Stage, Position, Stage × Position) with Benjamini–Hochberg adjustment and marginal R^2 for each alpha diversity index in bacteria and fungi.

Table S6. PERMANOVA and multivariate dispersion results: omnibus and pairwise PERMANOVA under permutation schemes respecting the paired design (Stage tested by permuting intact logs; Position and the interaction by within-log swaps; pairwise tests by

exact enumeration of permissible permutations), together with betadisper homogeneity-of-dispersion tests.

Table S7. ALDEx2 differential abundance summary: effect sizes (standardized median difference), 95% Monte Carlo intervals, and FDR values (we.eBH) for all 16 pairwise comparisons, with the genera passing the significance thresholds.

Table S8. Enrichment group assignments: genus-level membership of each bacterial $B-EG-I, B-EG-III, B-EG-V$ and fungal $F-EG-I, F-EG-III, F-EG-V$ enrichment group, together with the differential-abundance comparisons supporting each assignment.

Table S9. Cross-kingdom network statistics. Section A: StARS stability values and lambda selection for the three networks. Section B: netCompare permutation tests between the paired Internal and External networks. Section C: enrichment-group participation in the three networks. Section D: connector and hub role assignments (Zi–Pi). Section E: genus-by-genus detection, number of positive samples, and pooled relative abundance of enrichment-group members in each network sample set.

Table S10. Mantel tests between microbial community structure and wood chemistry. Part A: whole-community associations under three permutation schemes (within-stage, within-log swap, free permutation) with Benjamini–Hochberg adjustment. Part B: the same analysis at the enrichment-group level, reported as a characterisation of the groups.

Table S11. PLS-SEM statistics. S11a: measurement model (outer loadings, AVE, composite reliability); S11b1: cross-loading matrix; S11b2: HTMT ratios; S11c: variance inflation factors on latent variable scores; S11d0: model fit (GoF, R^2); S11d: structural path estimates

with cluster-bootstrap 95% confidence intervals; S11e: direct, indirect, and total effects; S11f: sensitivity analyses (within-log difference models, PCoA2-outcome models, row-wise bootstrap comparison); and the VIF fallback report for the pre-registered reduced models.

Table S12. BioSample and SRA run accessions: per-sample NCBI BioSample and SRA run accessions for the 16S rRNA and ITS1 amplicon sequencing data (BioProject PRJNA1499194).

Table S13. Functional assignments for enrichment-group genera: FUNGuild guild and confidence assignments (fungi), FAPROTAX functional group predictions (bacteria).

Figures

Figure S1. Linear mixed model R^2 for wood physicochemical properties: variance explained by the Stage × Position models for each of the eight measured properties (model statistics in Table S2).

Figure S2. Temporal and spatial dynamics of community composition: relative abundances of bacterial genera and fungal phyla across decay stages (I, III, V) and positions (Internal, External).

Figure S3. MA plots for the four differential abundance comparisons featured in Figure 2 (ALDEx2; statistics in Table S7).

Figure S4. Volcano plots for the remaining twelve differential abundance comparisons not shown in Figure 2 (ALDEx2; statistics in Table S7).

Figure S5. Enrichment-group Mantel associations with wood chemistry: group-level Bray–Curtis dissimilarity versus chemical distances for the bacterial and fungal enrichment groups, with

link width proportional to |Mantel *r*| and significance assessed under the three permutation schemes (Table S10, Part B). Because group membership was defined by decay-stage enrichment, these associations are expected by construction and are reported as a characterisation of the groups.

Figure S6. PLS-SEM total effects in the pre-registered reduced models: standardized total effects on community composition in the reduced (no-lignin) models M1 (bacteria) and M2 (fungi), for comparison with the full-model estimates in Figure 4d, e (statistics in Table S11).

Figure S7. PLS-SEM direct, indirect, and total effect decomposition: effects of the three exogenous constructs (Stage III, Stage V, Position) on community composition in models M1 and M2, showing the direct and chemistry-mediated (indirect) components underlying the total effects (statistics in Table S11e).
